# Defect Engineering to Achieve Photostable Wide Bandgap Metal Halide Perovskites

**DOI:** 10.1021/acsenergylett.3c00610

**Published:** 2023-05-31

**Authors:** Samuele Martani, Yang Zhou, Isabella Poli, Ece Aktas, Daniele Meggiolaro, Jesús Jiménez-López, E Laine Wong, Luca Gregori, Mirko Prato, Diego Di Girolamo, Antonio Abate, Filippo De Angelis, Annamaria Petrozza

**Affiliations:** † Center for Nano Science and Technology @Polimi, Istituto Italiano di Tecnologia, via Rubattino 81, 20134 Milano, Italy; ‡ Department of Chemical, Materials and Production Engineering, University of Naples Federico II, Piazzale Vincenzo Tecchio, 80, 80125 Napoli, Italy; § Computational Laboratory for Hybrid/Organic Photovoltaics (CLHYO), Istituto CNR di Scienze e Tecnologie Chimiche “Giulio Natta” (CNR-SCITEC), Via Elce di Sotto, 8, 06123 Perugia, Italy; ∥ Materials Characterization Facility, Istituto Italiano di Tecnologia, via Morego 30, 16163 Genova, Italy; ⊥ Department of Chemistry, Biology and Biotechnology, University of Perugia and INSTM, Via Elce di Sotto 8, I-06123, Perugia, Italy; # Department of Natural Sciences & Mathematics, College of Sciences & Human Studies, Prince Mohammad Bin Fahd University, Dhahran 34754, Saudi Arabia; ¶ SKKU Institute of Energy Science and Technology (SIEST) Sungkyunkwan University, Suwon 440-746, Korea

## Abstract

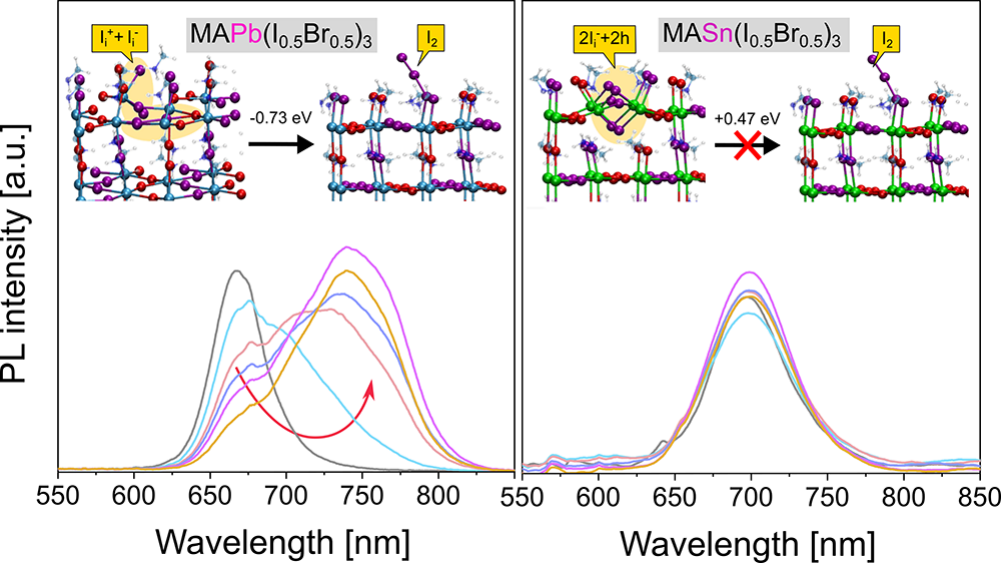

Bandgap tuning is a crucial characteristic of metal-halide perovskites, with benchmark lead-iodide compounds having a bandgap of 1.6 eV. To increase the bandgap up to 2.0 eV, a straightforward strategy is to partially substitute iodide with bromide in so-called mixed-halide lead perovskites. Such compounds are prone, however, to light-induced halide segregation resulting in bandgap instability, which limits their application in tandem solar cells and a variety of optoelectronic devices. Crystallinity improvement and surface passivation strategies can effectively slow down, but not completely stop, such light-induced instability. Here we identify the defects and the intragap electronic states that trigger the material transformation and bandgap shift. Based on such knowledge, we engineer the perovskite band edge energetics by replacing lead with tin and radically deactivate the photoactivity of such defects. This leads to metal halide perovskites with a photostable bandgap over a wide spectral range and associated solar cells with photostable open circuit voltages.

Metal-halide perovskites (MHPs) are wonder semiconductors endowed with chemical flexibility associated with a variety of optoelectronic properties, such as the possibility of tuning their bandgap.^1^ This allows creating a wide library of materials to be integrated in diversely purposed devices. An enormous impact is expected by the development of perovskite/silicon and perovskite/perovskite tandem solar cells, which require stable MHPs with a bandgap of >1.7 eV.^2,3^ However, this is actually a daunting task: the typical substitution of iodide with bromide in the MHP lattice can not only promote the bandgap over 1.7 eV but also introduces the instability of light-induced halide segregation, ultimately leading to the formation of iodide-rich and bromide-rich phases.^4^ Earlier reports discussed about a defect-driven phenomenon for this unusual behavior, which is consistent with the well-known facile ion migration repeatedly observed in MHPs.^5−9^ Confirming the defect-induced nature of the process, crystallinity improvement and surface passivation delivering high quality polycrystalline thin films (with expected lower defect densities) have indeed shown a reduced tendency of mixed-halide perovskites toward halide segregation.^10−12^ Significantly, these light-induced ion migration phenomena are an intrinsic characteristic of both pure-iodide and mixed-halide lead perovskites, showing up in the former as light-induced decomposition of the thin film,^13−16^ while in the latter they give rise to the spectacular bandgap variation following halide demixing.^6,8,9,17−19^

We investigated mixed-halide lead perovskites and exploited a well-known strategy for improving crystallinity and enlarging the grain size of the polycrystalline thin film (i.e., use of Pb(SCN)_2_-containing additive) which slows down the halide photosegregation.^20,21^ This allowed us to isolate and characterize the charge carrier dynamics associated with bandgap (de) stabilization. The electronic trap states and the associated defects that trigger halide demixing are identified, discovering that light-induced instabilities in pure-iodide and mixed-halide lead perovskites have a common origin. While the defect density can be minimized by ad hoc passivation or crystallinity improvement strategies, the entire suppression of defects is hard, being related to a thermodynamic property of the material. Thus, armed with the knowledge acquired on the electronic activity of such defect, we rather propose a strategy to inactivate it by shifting the band edge of the MHP to a region where the electronic state associated with the defect moves outside the semiconductor bandgap. We attained this by entirely replacing lead with tin, where the energy bands upshift related to the different metal orbital states eventually allow us to achieve photostable wide bandgap MHPs.

The Cs_0.17_FA_0.83_Pb(I_0.5_Br_0.5_)_3_ [FA = HC(NH_2_)_2_^+^] perovskite is first investigated, which has an ideal bandgap of 1.85 eV for the top absorber in a perovskite/perovskite tandem.^22^ The perovskite thin films are prepared with 0 to 5 mol % Pb(SCN)_2_-containing additive in the precursor solution (see Methods in the Supporting Information for details). The average grain size is greatly enlarged from ∼200 nm to ∼2 μm by increasing the quantity of Pb(SCN)_2_ from 0 to 5 mol %, as shown by the scanning electron microscopy (SEM) images in Figures 1a-b and S1a-e. The grain size enlargement matches with the highly enhanced crystallinity, as indicated by the increase in the intensity of (001) main X-ray diffraction (XRD) peak (Figures 1c and S1f-g) of the perovskite phase. The improvement in grain size and crystallinity is possibly due to the delayed thin film crystallization induced by Pb(SCN)_2_.^23^ We further explore the surface chemical environment of the thin films prepared without and with the additive by conducting the X-ray photoemission spectroscopy (XPS) measurements. Figure 1d shows the XPS peaks of Cs 3d, I 3d, N 1s, S 2p, Pb 4f, Cs 4d, and Br 3d. The XPS results show the presence of SCN^–^ on the surface of the perovskite film prepared with SCN^–^ addition because of the emergence of an additional N 1s peak at 398.3 ± 0.2 eV and S 2p doublet with S 2p_3/2_ and S 2p_1/2_ peaks at 162.7 ± 0.2 and 163.9 ± 0.2 eV, respectively, belonging to SCN^–^.^24^ The relative atomic concentration of each element is calculated from the area of the XPS peaks, after normalization to the relative sensitivity factors for each photoemission process, and the results are summarized in Table 1. In the perovskite prepared without additive, the stoichiometry obtained via XPS is Cs_0.17_FA_0.83_PbBr_1.48_I_1.54_ by taking the Pb concentration as a reference, which is in good agreement with the expected chemical formula of the perovskite. In the perovskite prepared with Pb(SCN)_2_, the relative concentrations of Cs, N in FA, Pb, Br and I decreased because of the presence of SCN^–^. The concentration of N in SCN^–^ groups is 4.1 at%, in good agreement with the S concentration (3.9 at%) calculated from the peak in the S 2p region, which further confirms the assignment of these two signals to the SCN moiety. Even though the concentrations of Cs and N from FA decrease slightly in the perovskite with SCN^–^, their ratios with reference to Pb remain unchanged (Table 1). Besides, the [I]/[Pb] only shows a slight decrease from 1.54 to 1.51. In contrast, the [Br]/[Pb] ratio drops significantly from 1.48 to 1.23, along with the increase of [SCN]/[Pb] ratio from 0 to 0.24–0.25. These results suggest that SCN^–^ most likely substitutes Br^–^ in the perovskite with SCN^–^. It must be noted that XPS measurement provides a surface-related compositional information, being the penetration depth of the technique of about 10 nm. Meanwhile, the XRD peaks show barely any shift of the main perovskite XRD peaks when Pb(SCN)_2_ is used (Figures 1c and S1f-g), suggesting that SCN^–^, which has an ionic radius of 213 pm,^25^ has negligible occupancy in the perovskite lattice (I^–^ and Br^–^ radii are 220 and 196 pm, respectively).^26^ Combining XPS with XRD results, we suggest that almost all the residual SCN^–^ ions in the perovskite substitute Br^–^ on the grain surface.

**Figure 1:**
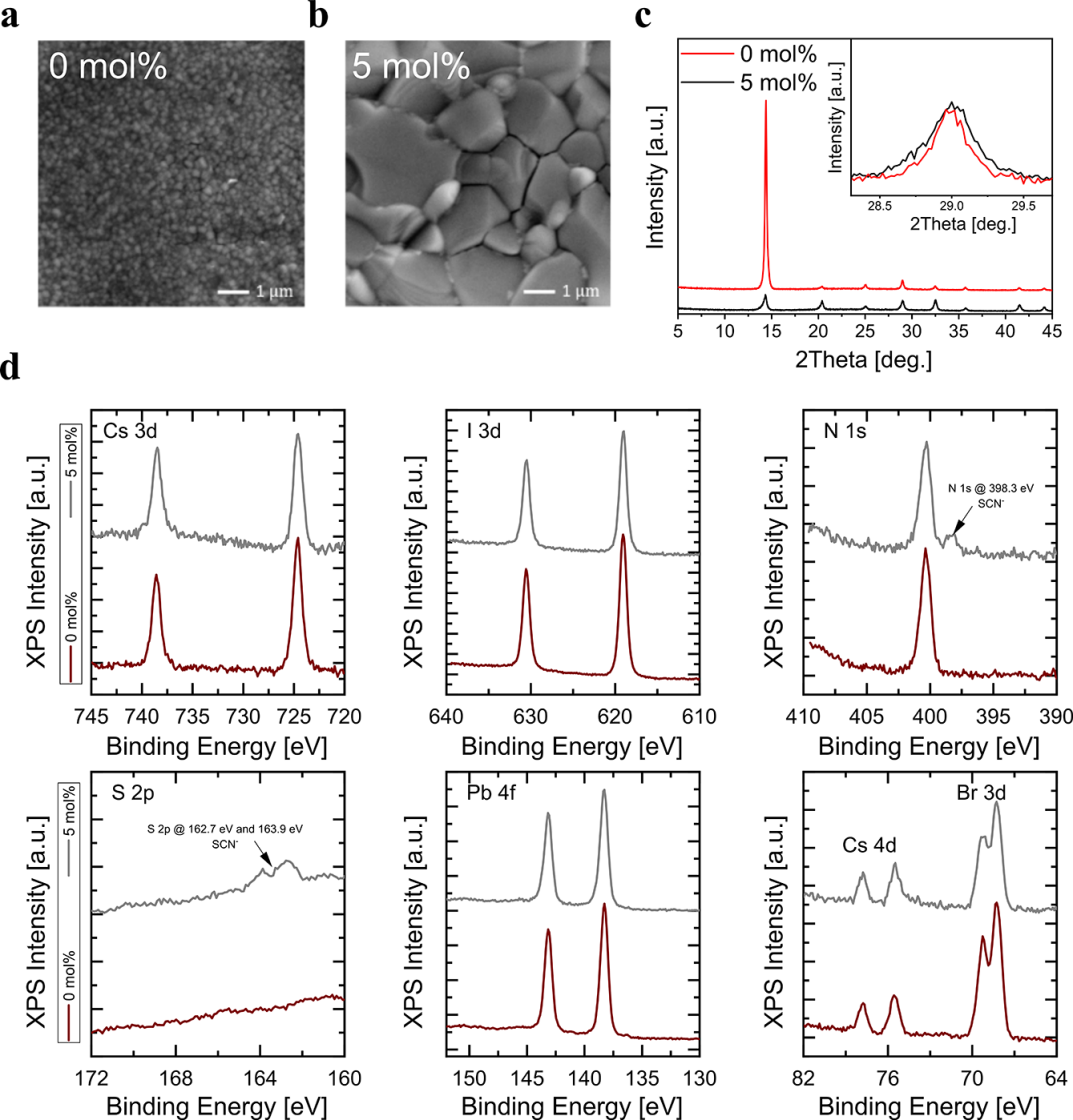
Impact of using the Pb(SCN)_2_-containing additive on morphology, crystallinity, and surface chemical environment of the perovskite. Top-view SEM images of the Cs_0.17_FA_0.83_Pb(I_0.5_Br_0.5_)_3_ perovskite thin films prepared a) with 0 mol % and b) 5 mol % addition of Pb(SCN)_2_. c) XRD patterns of the perovskite thin films with 0 and 5 mol % addition of Pb(SCN)_2_. The inset shows the (002) peak of the perovskite phase. d) XPS spectra of the Cs 3d, I 3d, N 1s, S 2p, Pb 4f, and Br 3d core levels of perovskite films processed with 0 and 5 mol % addition of Pb(SCN)_2_.

**Table 1:** Elemental Concentrations Derived from XPS Spectra

		Cs	N (from FA)	N (from SCN)	Pb	Br	I	S
Pristine	at%	2.9	28.5	0	17.1	25.3	26.3	0
	Ratio w. r. t. Pb	0.170	1.667	0	1	1.480	1.538	0
With SCN^–^	at%	2.8	27.5	4.1	16.5	20.3	24.9	3.9
	Ratio w. r. t. Pb	0.170	1.667	0.248	1	1.230	1.509	0.236

Figures 2a-b show the evolutions of the photoluminescence (PL) spectra of both the pristine reference (prepared without additive) and the additive containing (prepared with 5 mol % Pb(SCN)_2_) samples by a 450 nm diode laser (50 mW/cm^2^), operating in a continuous mode for 2 min. With this photon dose, the photoinduced halide segregation is prominent in the pristine reference sample, which manifests itself with the appearance of an additional red-shifted PL peak (Figure 2a).^4^ This is due to the funnelling of photoexcited carriers into the formed iodide-rich regions, which exhibits a lower bandgap than the starting mixed material^5^ (1.61 vs. 1.85 eV, respectively). In the additive containing sample, as expected,^20^ halide segregation is lifted out on the investigated time scale (Figure 2b). Thus, we take this model system to identify the optoelectronic processes associated with the observed phenomenology. The transient photocurrent (TPC) is first measured for both samples before halide segregation occurs (see PL spectra tracing during the experiments in Figure S2a). By monitoring TPC, we are not limited to radiative recombination but any free carrier dynamics in the semiconductor can be monitored.^27,34^ The TPC trace of the reference sample shows that part of the carrier population decay in the ns time window, while part of the population has an extremely slow dynamic (>10 μs) (Figure 2c), which is not observed by monitoring its band-to-band radiative recombination via time-resolved PL (TRPL) (Figure S2b). This implies that long-lived mobile photocarriers do not relax radiatively. Thus, the observed dynamics in TPC are associated with a long-lived trapped carrier,^27,34^ which disappears in the additive containing sample showing suppressed halide segregation.

**Figure 2:**
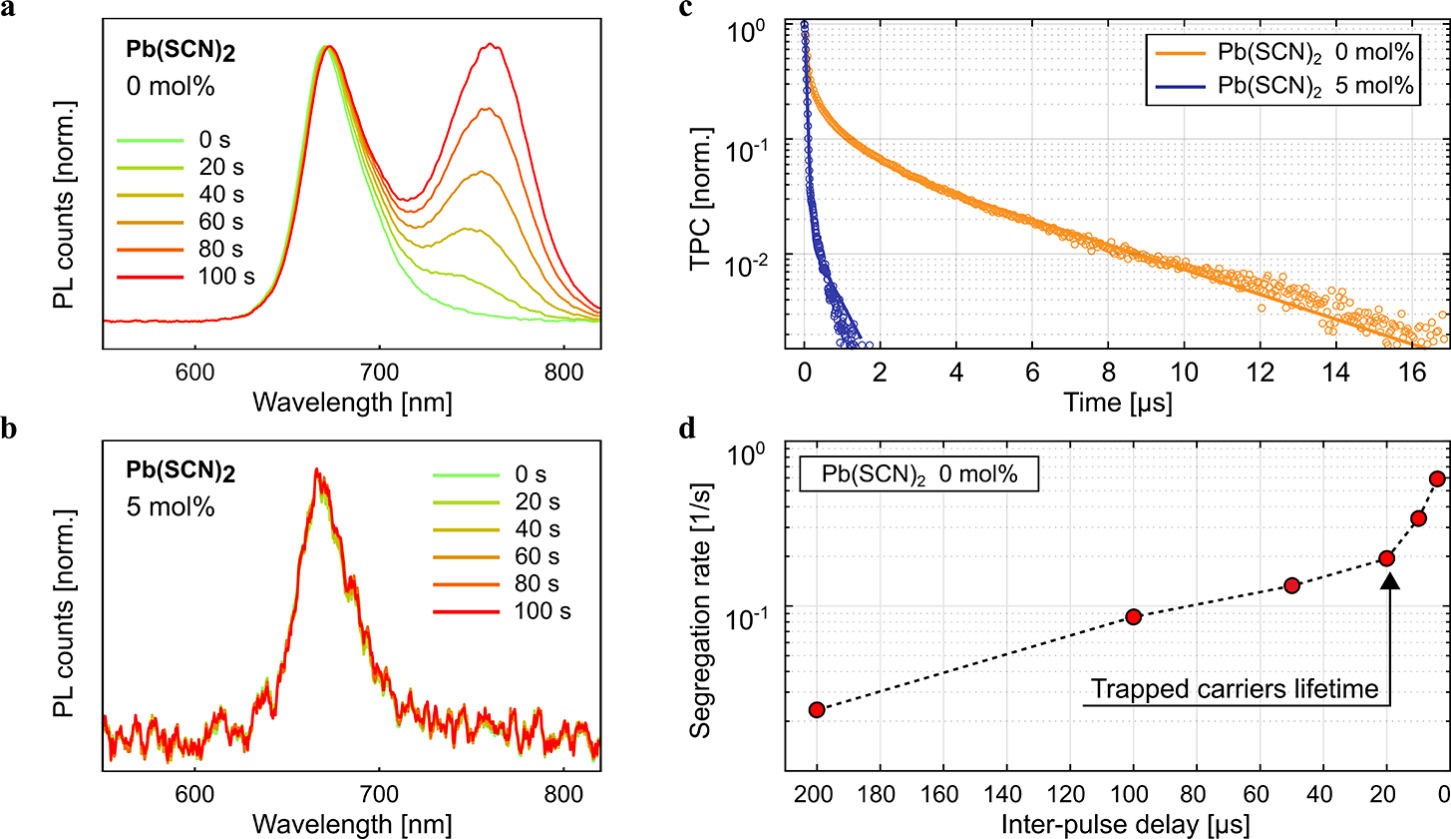
Spectroscopy analysis of Cs_0.17_FA_0.83_Pb(I_**0.5**_Br_0.5_)_3_perovskites. Evolutions of the PL spectra of the perovskite samples prepared a) without and b) with Pb(SCN)_2_ under a 450 nm diode laser (50 mW/cm^2^) over 2 min. The spectra are normalized at 670 nm. c) TPC kinetics of the perovskite samples prepared without and with Pb(SCN)_2_. d) Variations of halide segregation rate over interpulse delay in the sample prepared without Pb(SCN)_2_. The segregation process accelerates when the delay between pulses becomes comparable with the lifetime of long-lived trapped carriers (∼20 μs) estimated from the TPC tail (see Figures S4–S6).

The same experiment is also carried out on pure-iodide Cs_0.17_FA_0.83_PbI_3_ thin films, synthesized without and with Pb(SCN)_2_. Identical to the mixed halide scenario, the long-lived dynamics observed in the TPC trace disappears after using the additive in film preparation (Figure S3a). This observation is further consolidated by monitoring the photobleaching dynamics of the Cs_0.17_FA_0.83_PbI_3_ thin films prepared without and with Pb(SCN)_2_ via transient absorption (Figure S3b). Under-coordinated iodine defects, e.g., iodine interstitials and lead vacancies, were previously identified as photochemically active deep traps in MAPbI_3_.^27,28^ The peculiar iodine redox chemistry leads to extremely long (>μs) deactivation kinetics of slowly trapped electrons on positively charged interstitial iodine (I_i_^+^, basically a coordinated I_3_^–^ moiety corresponding to oxidized iodine),^27^ while negative interstitials (I_i_^–^, split iodine anions) and lead vacancies (featuring similar features as I_i_^–^) show a fast hole trapping-recombination, that quickly deplete the carrier population in the semiconductor. Carrier trapping at such defects leads to the formation of neutral I_i_^0^ species (basically, a coordinated I_2_^–^ radical) whose lifetime mirrors that of trapped carriers. The long lifetime of the I_2_^–^ radical species (when electrons are trapped) leads to a significant probability of forming I_2_ moieties through bimolecular processes (e.g., 2I_2_^–^ → I_2_ + 2I^–^) or capture of a second carrier (e.g., I_2_^–^ + 1h→ I_2_); I_2_ can be eventually expelled to the surface and grain boundaries, activating material degradation in the pure-iodide perovskite.^13^

Overall, based on the observations collected from both mixed halide and pure iodide model systems, we can conclude that the trapping of carriers at interstitial defects which eventually lead to the formation of I_2_ molecules is associated with the halide segregation process. Thus, we can also claim a common origin for phase segregation in mixed-halide perovskites and photodegradation in pure-iodide perovskites. It is rooted in the formation and diffusion of I_2_ thanks to the presence of long-lived dynamics of trapped carriers with a sufficiently long lifetime compatible with ionic dynamics and material transformation.

The direct connection between the defect activity linked to the long-lived carrier trap and photoinduced halide segregation can be pinned up by monitoring the evolutions of the PL spectrum of the mixed-halide reference sample under a pulsed laser with varied interpulse delay (Figures 2d and S4). The halide segregation rate at each interpulse delay is derived based on the evolution of the PL spectrum, and the method is described in the Supporting Information (Figure S4). The sub-bandgap feature of the segregated phase only becomes prominent for interpulse delays <20 μs, i.e., when the subsequent excitation pulse reaches the sample at a delay matching the lifetime of the long-lived traps (Figures 2c-d and S5). Accordingly, halide segregation rate shoots up super linearly when, upon photoexcitation, the species formed by carrier trappings start piling up in agreement with bimolecular/bielectronic reactions (Figure 2d). In the sample prepared with Pb(SCN)_2_, where the long-lived carrier trap dynamics is virtually not observed, bandgap instabilities do not appear even at a short interpulse delay of 2 μs (Figure S6).

We have so far identified the electronic dynamics and the related halide defect chemistry responsible for bandgap destabilization in lead halide perovskites. While this may support the development of a targeted strategy for crystallinity improvement/surface passivation, it also highlights the intrinsic instability related to halide photochemistry. Based on this picture, we propose a radical but viable solution that can entirely deactivate photochemically active halide defects, instead of reducing them, by pushing the perovskite band edges toward higher energy levels. One effective approach to push the valence band edge upward, consists in substituting tin for lead in the mixed halide perovskite lattice.

Since the peculiar halide photochemistry in conjunction with low ion migration barriers plays a major role in the phase segregation process,^6^ the defect activities of interstitial halides are investigated in lead mixed-halide perovskite (MAPbI_1.5_Br_1.5_) and in the corresponding tin counterpart (MASnI_1.5_Br_1.5_) by hybrid density functional theory (DFT) calculations. We focus on MA-based perovskites, rather than the experimentally employed CsFA compounds, for simplicity, considering the exiguous impact of A-site cations on the perovskite defect chemistry. We calculate defect formation energies (DFEs) and ionization levels of halide interstitials in both types of perovskites. As anticipated, our calculations show an increase of the valence band (VB) energy, i.e., a decrease of the ionization potential, of ∼0.9 eV in the tin perovskite compared to the lead counterpart (Figures 3a-b). The calculated bandgaps of the mixed-halide lead and tin perovskites are 1.98 and 1.85 eV, respectively, consistent with experimental PL maxima (∼1.85 and 1.63 eV, respectively, see Figures 2a and 4b).

**Figure 3:**
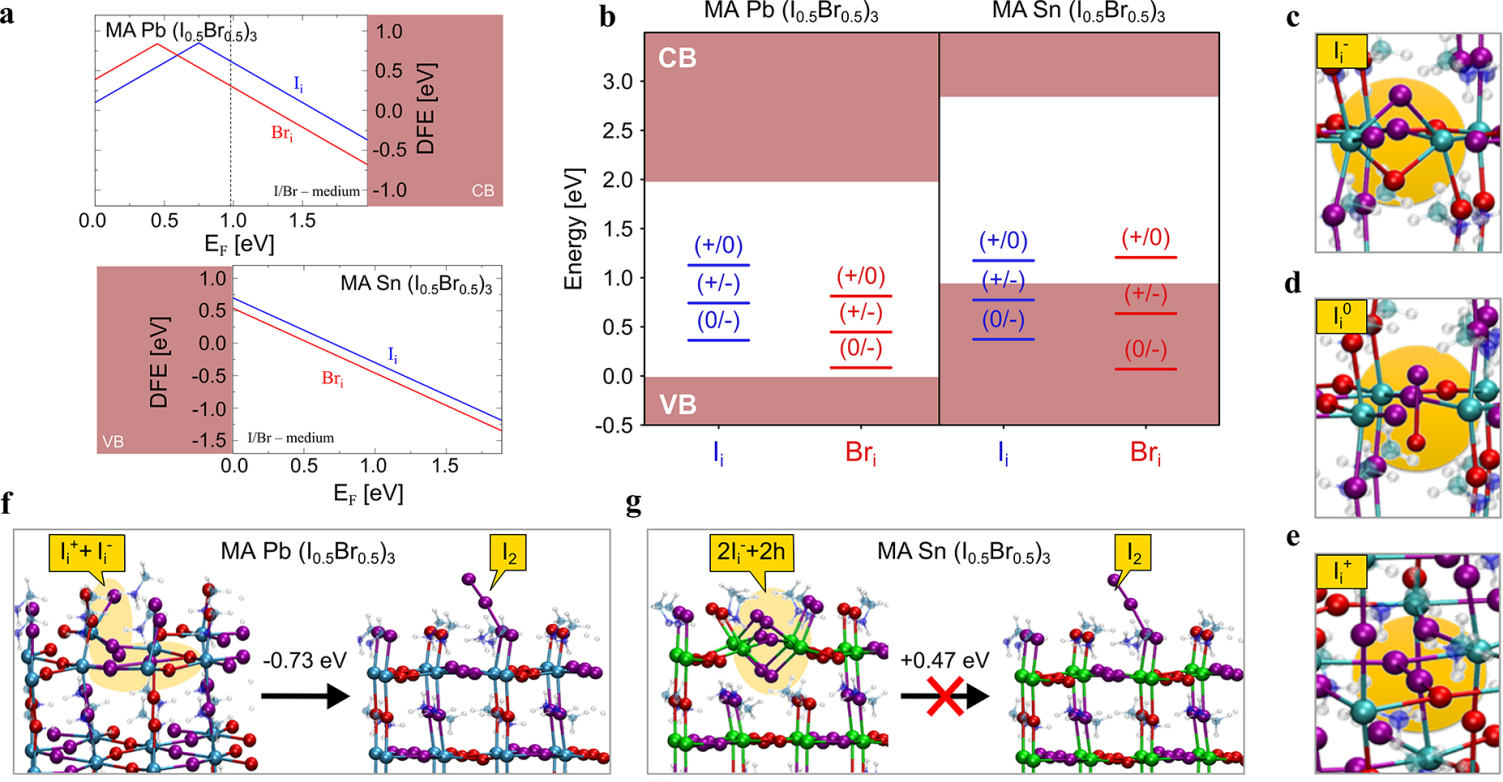
Simulation of trap activity in Pb-based and Sn-based mix-halide perovskites. a) Defect formation energies of iodine and bromine interstitials in bulk MAPb(I_0.5_Br_0.5_)_3_ and MASn(I_0.5_Br_0.5_)_3_ simulated in I/Br medium conditions (the band gaps of the perovskites have been corrected by rigidly applying spin orbit coupling corrections to the PBE0 band gaps). b) Thermodynamic ionization levels of the modeled halide interstitials. Equilibrium structures of c) negative, d) neutral, and e) positive iodine interstitial in MAPb(I_0.5_Br_0.5_)_3_. Equilibrium structures and relative energies of an I_2_ molecule adsorbed on the (001) MAI-terminated surface and the molecule incorporated into the slab to form split interstitial defects in f) MAPb(I_0.5_Br_0.5_)_3_ and g) MASn(I_0.5_Br_0.5_)_3_.

**Figure 4:**
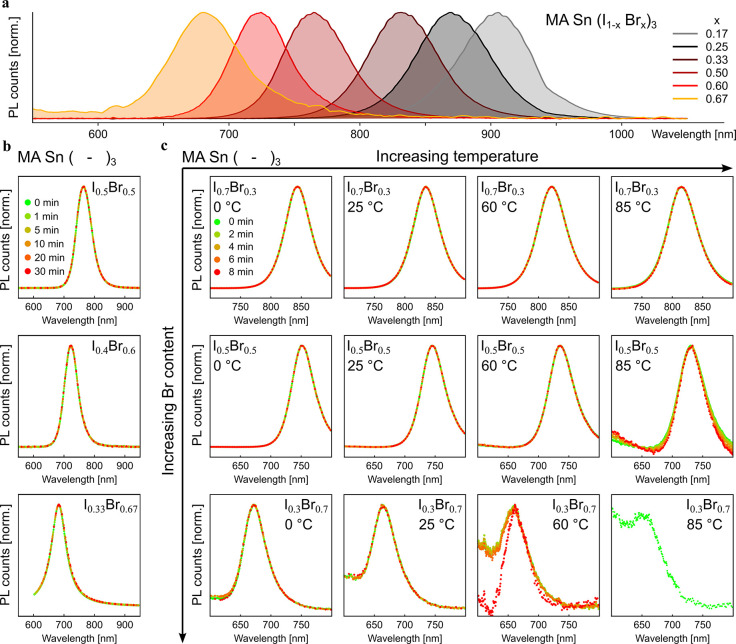
Photostability tests of Sn-based mix-halide perovskites. a) Steady-state PL spectra of MASn (I_1–*x*_Br_*x*_)_3_ perovskites at increasing bromide content. b) PL monitored under a 450 nm laser diode (50 mW/cm^2^) of MA-based perovskites with increasing bromide content. For each sample, the PL spectrum does not change during the duration of the experiment. c) PL monitored under a 530 nm laser diode (30 mW/cm^2^) of MA-based perovskites with increasing bromide content and temperature. Poor emissivity at high temperature prevented the execution of the experiment for perovskites with an iodide to bromide ratio of 0.3:0.7 at 85 °C.

The equilibrium structures of halide interstitials in various charge states of MAPb(I_0.5_Br_0.5_)_3_ closely resemble those in the pure MAPbI_3_^27^ (Figures 3c-e); both I_i_^+^ and Br_i_^+^ are stable in a relatively wide Fermi level range, up to 0.75 and 0.45 eV above the VB maximum (VBM), respectively. Notably, positive Br_i_^+^ tends to bind two lattice I^–^ ions to form an I–I–Br trimer with Br–I and I–I bond distances of ∼2.8 Å, clearly indicating the oxidation of a lattice iodide instead of the bromide interstitial. The lower stability of the oxidized Br_i_^+^ species with respect to I_i_^+^ is in line with the higher oxidation potential of bromide (1.07 V) compared to iodide (0.54 V), making bromide oxidation more energetically demanding. Iodine can indeed be selectively expelled from mixed-halide lead perovskites upon electrochemical bias.^29^ I_i_^+^ can trap electrons through the (+/0) transition while I_i_^–^ can trap holes through the (−/0) transition placed, respectively, at 1.13 and 0.37 eV above the VBM (Figure 3b). In both cases, trapping leads to the formation of the long-lived I_2_^–^ dimer species, which may further react leading to the formation of I_2_.^27^ Br_i_^–^, on the other hand, can trap holes through the shallow (0/−) transition located at 0.09 eV above the VBM (Figure 3b) in line with the higher bromide stability discussed above.

The defect chemistry of MASn(I_0.5_Br_0.5_)_3_ is remarkably different. The higher VB energy of the tin perovskite, i.e. its lower ionization potential, prevents the formation of positive halide interstitials and only negative interstitials are thermodynamically stable across the Fermi level range spanned by the bandgap, leading to thermodynamic transitions resonant with the VBM (Figures 3a-b).

The deactivation of trapping activities of halide interstitials by tin substitution suggests that iodide oxidation may only be active in lead but not in tin halide perovskites. This is confirmed by investigating the energetics of forming a I_2_ molecule through carrier trappings at iodine defects, followed by its exclusion to the surface and forming a trimer with a terminal iodide in both lead and tin halide perovskites^30^ (Figures 3f-g). In MAPb(I_0.5_Br_0.5_)_3_ the split I_i_^+^ + I_i_^–^ couple, i.e. the stable form of I_2_ in the bulk lattice, is 0.73 eV less stable than I_2_ adsorbed at the surface (Figure 3f), highlighting that I_2_ formation and expulsion at the surface is thermodynamically favored. On the contrary, in MASn(I_0.5_Br_0.5_)_3_, the surface adsorbed I_2_ molecule is only metastable, being 0.47 eV less stable than 2I_i_^–^ + 2h^+^ (Figure 3g), indicating that I_2_ formation and expulsion is not thermodynamically viable anymore due to the higher VBM which stabilizes holes residing in the VB.

In agreement with the results of DFT calculations, the I_2_ formation and expulsion is not detected when the MASn(I_0.5_Br_0.5_)_3_ perovskite film is kept under a continuous white LED (100 mW/cm^2^) for about 30 h (Figures S7a-b). Importantly, we did not probe any change in the dark conductivity of the tin halide perovskite before and after illumination (Figure S8). On the other hand, I_2_ is detected when the same test is performed on Cs_0.17_FA_0.83_Pb(I_0.5_Br_0.5_)_3_ thin films (Figure S7b). Most importantly, Sn-perovskites do not show I_2_ formation even when the volatile MA cation is used, which is known to lead to thin films with lower optoelectronic quality than Cs-containing compositions.^31^ It is also found that the lead based perovskite Cs_0.17_FA_0.83_Pb(I_0.5_Br_0.5_)_3_ prepared with Pb(SCN)_2_ shows much reduced I_2_ formation, in agreement with its reduced halide demixing discussed above.

Furthermore, we fabricated MASn(I_1–*x*_Br_*x*_)_3_ and FASn(I_1–*x*_Br_*x*_)_3_ thin films with *x* ranging between 0.17 and 0.67 (top-view SEM images of the films are shown in Figures S9–10) and checked their photostability. As expected, films prepared with higher bromide content show a significant blueshift of the PL emission, allowing for the fabrication of tin perovskite films with bandgaps tuned over the 1.37–1.82 eV range (Figures 4a and S11a). We monitored the evolutions of the PL of all samples under continuous illumination (50 mW/cm^2^ provided by a 450 nm diode laser) for up to 30 min (Figures 4b, S11b-d and S12). The PL spectra of all films are remarkably stable, even when they are excited with very high excitation intensities of 500 mW cm^–2^ (Figure S13). Even materials with high bromide fraction do not show bandgap changes over time, independently of the cation composition. Temperature may have a nontrivial effect on halide segregation: while a higher temperature favors entropically driven mixing,^32^ it can also provide the necessary activation energy to allow defect migration.^33^ We conducted a comprehensive temperature-dependent analysis of MASn(I_1–*x*_Br_*x*_)_3_. In Figures 4c and S14, we see that between 0 and 85 °C all perovskite compositions are stable under a 530 nm diode laser (30 mW/cm^2^).

Finally, we explored the impact of deactivation of halide segregation on the photostability of the tin-based mix-halide perovskite solar cells. The Cs_0.17_FA_0.83_SnI_1.5_Br_1.5_ solar cell is fabricated for the study, while the lead counterpart (Cs_0.17_FA_0.83_PbI_1.5_Br_1.5_ solar cell) is used as the reference (See Supporting Information for the fabrication detail). The photovoltaic parameters of the FA_0.83_Cs_0.17_PbI_1.5_Br_1.5_ and FA_0.83_Cs_0.17_SnI_I.5_Br_1.5_ solar cells, recorded with simulated AM1.5G illumination are shown in Figures S15–16. The in situ PL measurement at open circuit condition and under a 450 nm laser with an intensity of 50 mW/cm^2^ (Schematically shown in Figure 5a) are conducted for both types of solar cells under an inert N_2_ environment. This measurement allows us to simultaneously monitor changes in the open circuit voltage (*V*_OC_) of the solar cell and in the PL spectrum to directly correlate the potential effect that halide segregation and bandgap (in) stability has on the *V*_OC_. As shown in Figures 5b-c, the Pb-based perovskite embedded in a solar cell demonstrates fast halide segregation within only 5 min accompanied by a *V*_OC_ reduction of the cell under continuous excitation. In contrast, the PL spectra and *V*_OC_ of the Sn-based counterpart are extremely stable when tested for more than 30 h under the same condition (Figures 5d-e), confirming the excellent phase stability of Sn-based mixed-halide perovskite, enabling very stable *V*_OC_ of the working device.

**Figure 5:**
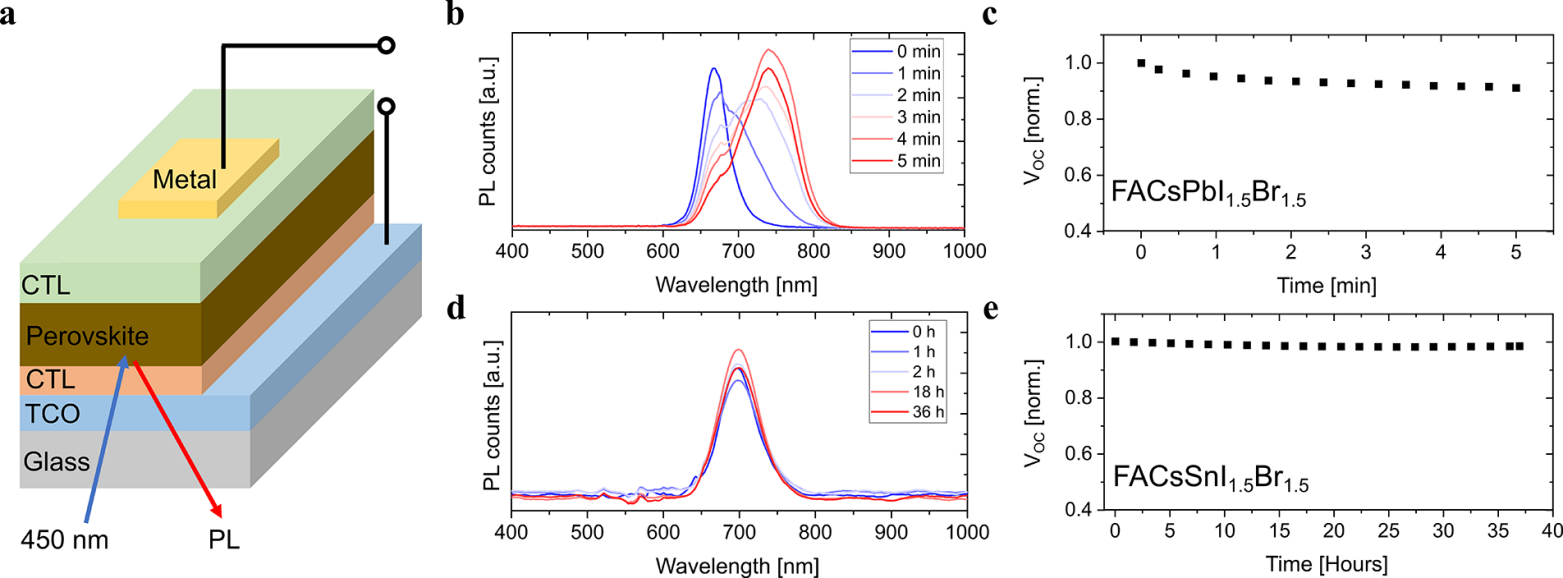
In situ PL and *V*_OC_ tracking of Pb-based and Sn-based mix-halide solar cells. a) Schematic of in situ PL measurement of the Pb-based and Sn-based mixed iodide-bromide perovskite solar cell at open circuit condition. The solar cells are illuminated by using a 450 nm diode laser with an intensity of 50 mW/cm^2^. TCO is transparent conductive oxide; CTL is a charge transporting layer. Time evolution of the b) PL and c) *V*_OC_ of FA_0.83_Cs_0.17_PbI_1.5_Br_1.5_ solar cell during the in situ PL measurement over 5 min, showing fast photoinduced halide segregation and *V*_OC_ decay. Time evolution of the d) PL and e) *V*_OC_ of FA_0.83_Cs_0.17_SnI_1.5_Br_1.5_ solar cell during the in situ PL measurement over 36 h, showing stable PL spectra and *V*_OC_ (pristine Sn perovskites prepared without any additive).

In conclusion, we have demonstrated that light-induced instabilities in pure-iodide and mixed-halide lead perovskites have common origin, being rooted in the photochemical activities of iodine-defects activated by the deep VB of the compounds. While in pure iodide lead perovskites iodide-defects cause decomposition,^16^ in mixed-halides they induce additional bandgap instabilities. Both degradation pathways can be slowed down, but not entirely suppressed, by using reducing agents and surface passivation treatments, indicating that they are intrinsic and thermodynamically favored phenomena. Surprisingly, tin halide perovskites, which are extremely sensitive to oxidation, turn out to be remarkably photostable, allowing us to fabricate wide bandgap materials with target characteristics for tandem solar cells. Key to such an achievement is the higher lying VB of tin halide perovskites than the lead counterparts, which, while on the one hand leads to a high propensity to tin oxidation, on the other hand destabilizes iodide oxidation and eliminates halide demixing. Overcoming thermodynamically favored light-induced instabilities by deactivation of defect activities through VB engineering is thus the key toward segregation-free mixed-halide perovskites.

## Supporting Information Available

Experimental section and additional characterization and discussion, including SEM images, XRD, XPS, steady-state and time-resolved PL, and current-density voltage curves (PDF)
