# Supplementary material for: Defect Engineering to Achieve Photostable Wide Bandgap Metal Halide Perovskites
Source: ACS Energy Lett. 2023 May 31;8(6):2801–8. doi: 10.1021/acsenergylett.3c00610 (PMC10262265; doi:10.1021/acsenergylett.3c00610)
Supplement: Supplementary file 1 — nz3c00610_si_001.pdf [file nz3c00610_si_001.pdf]

Supporting Information for

Defect Engineering to Achieve Photostable Wide Bandgap Metal Halide Perovskites

Authors: Samuele Martani[1]†, Yang Zhou[1]†*, Isabella Poli[1]†, Ece Aktas[2], Daniele Meggiolaro[3], Jesús Jiménez López[1], E Laine Wong[1], Luca Gregori[3,5], Mirko Prato[4], Diego Di Girolamo[2], Antonio Abate[2], Filippo De Angelis[3,5,6,7]*, Annamaria Petrozza[1]*

Affiliations:

[1]Center for Nano Science and Technology @Polimi, Istituto Italiano di Tecnologia; via Rubattino 81, 20134 Milano, Italy.

[2]Department of Chemical, Materials and Production Engineering, University of Naples Federico II; Piazzale Vincenzo Tecchio, 80, 80125 Napoli, Italy.

[3]Computational Laboratory for Hybrid/Organic Photovoltaics (CLHYO), Istituto CNR di Scienze e Tecnologie Chimiche 'Giulio Natta' (CNR-SCITEC); Via Elce di Sotto, 8, 06123 Perugia, Italy.

[4]Materials Characterization Facility, Istituto Italiano di Tecnologia; via Morego 30, 16163 Genova, Italy.

[5]Department of Chemistry, Biology and Biotechnology, University of Perugia and INSTM, Via Elce di Sotto 8, I-06123, Perugia, Italy.

[6]Department of Natural Sciences & Mathematics, College of Sciences & Human Studies, Prince Mohammad Bin Fahd University; Dhahran 34754, Saudi Arabia.

[7]SKKU Institute of Energy Science and Technology (SIEST) Sungkyunkwan University, Suwon, Korea 440-746.

*Corresponding authors. Email: yang.zhou@iit.it, filippo.deangelis@unipg.it, annamaria.petrozza@iit.it

Methods

Photoluminescence measurements under continuous light excitation (unless specified) by a 450 nm diode laser. The PL is acquired with a Maya 2000 pro spectrometer from Ocean Optics. The excitation intensity is adjusted depending on the experiment: for light-sensitive Pb-based mixed halide perovskite is set to 5 mW/cm^2 (equivalent to 0.1 sun) when recording the unperturbed emission spectrum, used for bandgap and PLQY estimation to 50 mW/cm^2 (equivalent to 1 sun) to evaluate the impact of halide segregation during light soaking. Sn-based mixed halide perovskite is characterised and lightly soaked at 1 Sun (or higher when specified) thanks to their higher photostability.

Transient absorption spectroscopy (TA) was collected in transmission geometry. An amplified femtosecond laser (Light Conversion Pharos) generated pulses of ~280 fs centred at 1030 nm. A broadband white light probe is generated by focusing the pulses into a thin sapphire plate. At short delays (<5 ns), the second harmonic of the fundamental provided the pump light (515 nm). At long delays (>1 ns), pump light at 532 nm was provided by the second harmonic of a Q-switched Nd: YVO4 laser (Innolas Picolo), which was electronically triggered and synchronized to the femtosecond laser via an electronic delay. The data acquired in the two-time regimes were combined, with a small scaling factor applied to overlap signal amplitudes between 2 and 4 ns. Kinetics are obtained by integrating over a wavelength window of 40 nm centred at the peak of the main photo-bleach (PB) at the band edge.

Transient Photo-Luminescence (TrPL) shares the same light excitation source as TA (second harmonic of the Pharos femtosecond laser 515 nm at 1 kHz repetition rate). This ensures that the data sets from the two techniques are collected at comparable excitation densities. The PL is coupled to an optical fiber, and a long-pass filter removes the pump laser scattering. An Andor spectrometer paired with an iStar iCCD is used for the detection. The camera acquisition is synchronized with the 1 kHz trigger signal from the laser. We track the photoemission dynamics by gating the camera's acquisition over a short temporal window (1 ns) collected at increasing delay from the trigger reference (1 ns steps). The overall time resolution of the system is 2-4 ns. Kinetics are obtained by integrating the whole PL spectrum recorded at each delay.

Transient Photo-Current (TPC) detects the relaxation of photocarriers generated by the second harmonic pulses of the Pharos femtosecond laser (515 nm) at a 1 kHz repetition rate (unless specified). Following the same sample architecture adopted by Leijtens et al [1]. (6 mm distance between the electrodes), we ensure an ohmic response limited by the semiconductor layer rather than the contacts. The laser spot was defocused to cover the gold contacts area fully. We used a trans-impedance amplifier (TIA) to measure the photo-current and to provide a 10 V bias to the sample (Femto DLPCA-100). An oscilloscope samples the voltage output from the TIA. The TIA bandwidth limits the experiment's time resolution at the selected gain ($V/A = 10^6$, time resolution of 0.2 μs).

Overlapping TA and TrPL kinetics requires that the two data sets are acquired at the same excitation density and wavelength. To achieve this, the pump laser from TA and excitation for TrPL are provided by the same laser and with the same optical path. Simply integrating the main photo-bleach signal (PB) in TA and the PL spectrum in TrPL doesn't produce comparable

relaxation kinetics. While TrPL depends on radiative carrier recombination for detecting a signal, TA is simply proportional to the presence of free carriers (which in perovskites cause a bleaching signal by blocking ground-state absorption). If we assume that electrons and holes generate the same contribution to ground state bleaching, the signal intensity for TA will be proportional to e(t) + h(t) (sum of the two separate carrier populations). The TrPL signal is instead proportional to e(t)*h(t). When the two carrier populations are similar, we can assume $e(t) \approx h(t) = n(t)$. Being TrPL $\propto n(t)^2$ and TA $\propto n(t)$, to correctly compare the two kinetics we need to square the values from the TA set of data. This assumption is valid at early times when radiative recombination and fast trapping are dominant and the two carrier populations remain balanced. Later, selective long-lived trapping of one of the two charge carriers can result in an unbalance between the two populations, and in that case we expect the two kinetics to diverge.

X-ray Photoelectron Spectroscopy (XPS) analyses were performed to evaluate the chemical composition of the materials under investigation. The measurements were carried out with a Kratos Axis Ultra^{DLD} spectrometer. High-resolution spectra were acquired at a pass energy of 10 eV using a monochromatic Al Kα source (15 kV, 20 mA), over the binding energy regions typical for Cs 3d, I 3d, N 1s, S 2p, C 1s, Pb 4f and Br 3d peaks.

SEM and XRD: Film morphology was characterized using scanning electron microscopy (SEM, Mira3, Tescan). X-ray diffraction (XRD) patterns of the samples were recorded using a Bruker D8 Advance equipped with a Cu Kα1 (λ = 1.544060 Å) anode, operating at 40 kV and 40 mA.

Voltage-Current measurements of the solar cells: The current density - voltage (J-V) characteristics of the solar cells were measured with a computer-controlled Keithley 2420 source meter in the air with device encapsulation. The simulated Air Mass 1.5 Global (AM 1.5G) irradiance was provided with a class AAA Newport solar simulator. The light intensity was calibrated with a silicon reference cell with a spectral mismatch factor of 0.99. The active area of the complete device was determined by an illumination-shadowing mask which is 0.0935 cm^2. For the Pb-based cells, the J-V measurement was done with a scan rate of 0.05 V/s, between 0 and 1.4 V. Preconditional stress was not done for PV measurements. Sn-based cells were tested by scanning between -0.1 and 0.6 V with a scan rate of 0.1 V/s.

In-situ PL measurement of the solar cells: To record PL and V_{OC} data simultaneously during continuous illumination (450 nm, 50 mW/cm^2) at open circuit condition, the solar cells are mounted in a commercial stability platform (ARKEO - Cicci Research) with N_2 flow (temperature ≈ 27 °C). All the cells were encapsulated before the stability test.

Iodine expulsion in hexane: Two equal cuts of the same perovskite sample are placed in two vials filled with hexane (4 ml). One vial is placed under 100 mW/cm^2 illumination (LED-based Solar Simulator (30x30cm) with illumination ranging from 0 to 2 sun (equivalent), 496 LED emitters with low mismatch 390-700 nm, Arkeo Cicci Research) for 30 hours, while the other is kept in dark. We can estimate the concentration of I_2 released by the sample into the solvent by measuring the absorption spectrum of the hexane taken from the vials. The presence of I_2 generates additional features between 400-600 nm in the hexane absorption spectrum[32]. All the displayed spectra are the result of the difference between the absorption spectra of the hexane taken from the

vials after the experiment and pristine hexane. The absorption spectra of the hexane from the vials kept in dark show the amount of I_2 released due to material degradation and in our case no discernible features are detected (dashed lines).

DFT simulations: the stability study of mixed $MAPb(I_{1-x}Br_x)_3$ and $MASn(I_{1-x}Br_x)_3$ perovskites has been carried out in the DFT framework by calculating the mixing Gibbs free energies of the phases for different I/Br ratios, see SI for results and details. Halide interstitial defects calculations have been carried out in the 2x2x2 tetragonal supercells of $MAPb(I_{0.5}Br_{0.5})_3$ and $MASn(I_{0.5}Br_{0.5})_3$, by using the CP2K code [2]. Cell parameters of the pristine phases have been fixed to the values obtained by the relaxation procedure used in the stability study. Calculations have been performed at the Γ point of the Brillouin zone (BZ) by using the hybrid PBE0 functional[3] and DFT-D3 dispersion corrections [4]. Norm-conserving Goedecker-Teter-Hutter pseudopotentials [5] and DZVP gaussian basis sets [6] have been used along with a density cutoff of 300 Ry on the charge density. To speed up hybrid functional calculations, the auxiliary density matrix method has been used [7].

The defects formation energies and the thermodynamic ionization levels have been calculated by using the following expressions [8, 9]

$$DFE\,[X^q] = E[X^q] - E[perf] - \mu_X + q(\varepsilon_{VB} + \varepsilon_F) + E^q_{\mathrm{corr}} \tag{3}$$

$$\varepsilon(q/q') = \frac{E[X^q] - E[X^{q'}]}{q' - q} + \frac{E^q_{corr} - E^{q'}_{corr}}{q' - q} - \varepsilon_{\mathrm{VB}} \tag{4}$$

where the term *E[X^q]* is the energy of the supercell with halide defect *X* in charge state *q*, *E(perf)* is the energy of the perfect supercell, μ_X is the chemical potentials of the halide species added to the perfect system, $\varepsilon_{\mathrm{VB}}$ is the valence band energy, ε_{F} and E_q^{corr} the Fermi level of the system and corrections due to periodic charges. Makov-Payne correction terms have been applied considering the static dielectric constants of $(MAPbI_{0.5}Br_{0.5})_3$ $\varepsilon_0 = 22$ and $(MASnI_{0.5}Br_{0.5})_3$ $\varepsilon_0 = 27$, estimated by averaging the values of the pure bromide and iodide phases. DFEs in Figure 3a have been calculated in I/Br medium conditions, as the intermediate chemical potentials between I/Br rich conditions and I/Br poor conditions. Specifically, for I/Br rich conditions the following chemical potentials have been used: for $MAPb(I_{0.5}Br_{0.5})_3$ $\mu(Br) = 1/2\ \mu(Br_2)$; $\mu(I) = 1/2\ \mu(I_2)$, with Br_2 and I_2 the isolated molecules); for $MASn(I_{0.5}Br_{0.5})_3$ by considering the equilibrium with the SnI_4 phase, i.e. $\mu(Br) = (\mu(SnBr_2)- 2\mu(SnI_2) + \mu(SnI_4))/2$; $\mu(I) = (\mu(SnI_4) - \mu(SnI_2))/2$. In I/Br poor conditions, for $MAPb(I_{0.5}Br_{0.5})_3$ $\mu(Br) = (\mu(PbBr_2) - \mu(Pb_{bulk}))/2$ and $\mu(I) = (\mu(PbI_2) - \mu(Pb_{bulk}))/2$ and for $MASn(I_{0.5}Br_{0.5})_3$ $\mu(Br) = (\mu(SnBr_2) - \mu(Sn_{bulk}))/2$ and $\mu(I) = (\mu(SnI_2) - \mu(Sn_{bulk}))/2$, with $\mu(Pb_{bulk})$ and $\mu(Sn_{bulk})$ the chemical potentials of bulk Pb and Sn metals.

I_2 molecule and defect couples at the surface (Figs. 3f-g) have been simulated in the supercell approach by using the PBE functional [10] and including DFT-D3 dispersion corrections.

Materials and Devices

Substrate cleaning. Fluorine-doped tin oxide (FTO)-coated glass was etched with zinc powder and 2 M aqueous HCl solution for electrode pattern. The FTO and glass substrates were

washed with 2% Hellmanex in water, deionized water, iso-propanol, acetone, and iso-propanol subsequently in a sonication bath for 15 min.

Pb-based mixed-halide perovskites: a solution of $Cs_{0.17}FA_{0.83}PbI_{1.5}Br_{1.5}$ $[(CsI)_{0.17}(FAI)_{0.83}(PbI_2)_{0.25}(PbBr_2)_{0.75}]$ (denoted as solution A) was prepared by mixing 0.17 M CsI (Alfa- Aesar, 99.999%), 0.83 M FAI (Great Solar), 0.25 M PbI_2 (TCI, 99.99%) and 0.75 M $PbBr_2$ (TCI, 99.99%) in anhydrous DMF: DMSO 4:1 (v:v) and stirred overnight. The $Pb(SCN)_2$ contained solutions of $(CsI)_{0.17}(FAI)_{0.83}[Pb(SCN)_2{+}2FAI]_{0.25}[Pb(SCN)_2{+}2FABr]_{0.75}$ (denoted as solution B) were prepared by mixing 0.17 M CsI (Alfa-Aesar, 99.999%), 1.33 M FAI (Great Solar), 1.5 M FABr (Great Solar) and 1 M $Pb(SCN)_2$ (Sigma-Aldrich, 99.5%) in anhydrous DMF: DMSO 4:1 (v:v) and stirred overnight. The following reaction happens by using the additive [11, 12]:

$$2Pb(SCN)_2 + 2FAX \rightarrow PbX_2 + FASCN\uparrow$$

Here X is iodide or bromide. In this way, using the additive will maintain the stoichiometry of the perovskite of $Cs_{0.17}FA_{0.83}PbI_{1.5}Br_{1.5}$. The precursor used for perovskite deposition was prepared by adding solution B with volume ratios of 0%, 1.25%, 2.5%, 3.75% and 5%. The precursor solutions were spin-coated onto the glass or FTO substrates in a two-step procedure at 1000 and 5000 rpm for 10 s and 40 s respectively. During the second step, 120 µL of chlorobenzene was casted onto the substrates 10 s before the ending of the second spinning step. The films were placed on a hotplate at 100 °C for 60 min.

$Cs_{0.17}FA_{0.83}PbI_3$ perovskite: a solution of $Cs_{0.17}FA_{0.83}PbI_3$ was prepared by mixing 0.17 M CsI (Alfa- Aesar, 99.999%), 0.83 M FAI (Great Solar) and 1 M PbI_2 (TCI, 99.99%) in anhydrous DMF: DMSO 4:1 (v:v) and stirred overnight. The $Pb(SCN)_2$ contained solutions of $(CsI)_{0.17}(FAI)_{0.83}[Pb(SCN)_2{+}2FAI]$ were prepared by mixing 0.17 M CsI (Alfa- Aesar, 99.999%), 2.83 M FAI (Great Solar) and 1 M $Pb(SCN)_2$ (Sigma-Aldrich, 99.5%) in anhydrous DMF: DMSO 4:1 (v:v) and stirred overnight. The precursor used for perovskite deposition was prepared by adding 5 vol% of $Pb(SCN)_2$-contained precursor into $Cs_{0.17}FA_{0.83}PbI_3$ precursor. The precursor solutions were spin-coated onto the glass or FTO substrates in a two-step procedure at 1000 and 5000 rpm for 10 s and 40 s respectively. During the second step, 120 µL of chlorobenzene was casted onto the substrates 10 s before the ending of the second spinning step. The films were placed on a hotplate at 100 °C for 60 min.

Sn-based mixed-halide perovskites: to make Sn-based $MASn(I_{1-x}Br_x)_3$ thin-films, the precursor solution was obtained by mixing varying ratios of equimolar solutions of (i) MAI + SnI_2 (1.2 M) and (ii) MABr and $SnBr_2$ (1.2 M). To make Sn-based $FASn(I_{1-x}Brx)_3$ thin-films, the precursor solution was obtained by mixing varying ratios of equimolar solutions of (i) FAI + SnI_2 (1.2 M) and (ii) FABr and $SnBr_2$ (1.2 M). All solutions were prepared using mixed solvents DMF:DMSO (4:1), stirred at 40°C for 30 min and then filtered through 0.20-µm PTFE membrane before use. The perovskite films were deposited with one-step spin-coating procedures at 4000 r.p.m. for 50 s. Anisole (80 µl) was dropped on the spinning substrate 25 s before the end of the procedure. The substrates were annealed at 100°C for 30 min.

Solar cells of Pb-based mixed-halide perovskites:

Preparation of electron transporting layer. The SnO_2 colloid precursor was obtained from Alfa Aesar (tin(IV) oxide, 15% in H_2O colloidal dispersion). Before use, the SnO_2 colloid precursor was mixed with water in a volume ratio of 1:5.67. The FTO substrates were treated by O_2 plasma cleaning for 10 min before depositing SnO_2. The diluted SnO_2 colloid solution was spin coated onto FTO substrates at 3000 rpm for 30 s, and then baked on a hotplate in ambient air (humidity

around 70% RH, temperature around 22 °C) at 120 °C for 10 min, followed by 30 min at 180 °C. After annealing, the hotplate was switched off, and the FTO substrates were allowed to cool down to room temperature naturally. Subsequently, the substrates were treated by UV-ozone for 15 min and then transferred to an inert N_2 filled glovebox.

Preparation of perovskite layer. A solution of $Cs_{0.17}FA_{0.83}PbI_{1.5}Br_{1.5}$ [$(CsI)_{0.17}(FAI)_{0.83}(PbI_2)_{0.25}(PbBr_2)_{0.75}$] (denoted as solution A) was prepared by mixing 0.17 M CsI (Alfa- Aesar, 99.999%), 0.83 M FAI (Great Solar), 0.25 M PbI_2 (TCI, 99.99%) and 0.75 M $PbBr_2$ (TCI, 99.99%) in anhydrous DMF: DMSO 4:1 (v:v) and stirred overnight. The precursor solutions were spin-coated onto the glass or FTO substrates in a two-step procedure at 1000 and 5000 rpm for 10 s and 40 s respectively. During the second step, 120 μL of chlorobenzene was casted onto the substrates 10 s before the ending of the second spinning step. The films were placed on a hotplate at 100 °C for 60 min.

Preparation of hole transporting layer and Au electrode. The hole-transport layer precursor was prepared by dissolving 72.3 mg spiro-MeOTAD (Luminescence Technology, 99.9%), 28 μL 4-tert-butylpyridine (Sigma-Aldrich, 96%) and 17.5 μL bis(trifluoromethane)sulfonimide lithium salt (Sigma-Aldrich) solution (520 mg Li-TFSI in 1 mL acetonitrile) in 1 mL chlorobenzene. The precursor was then spin-coated on the perovskite layer at 4000 rpm for 30 s. The deposition of the hole transport layer was in the glovebox. The solar cells were then transferred to a box with dry air (humidity lower than 20% RH) for oxidation for 16 h. Finally, a 100 nm Au electrode layer was deposited under a vacuum of $<1\times10^{-4}$ Pa with a rate of 0.1nm/s. The preparation of the Au electrode was performed in a N_2-purged glovebox.

Solar cells of Sn-based mixed halide perovskite

The indium tin oxide (ITO) coated glass substrates were sequentially cleaned in Hellmanex 2% deionised (DI) water solution, DI water, acetone, and 2-propanol by sonication at 40 °C for 15 min. The well-dried substrates were cleaned via UV-ozone treatment for 15 min.

Preparation of hole transporting layer. The PEDOT solution precursor was prepared by diluting PEDOT (HTL3 from Clevios) with toluene. ITO substrates were treated by O_2 plasma cleaning for 10 min before depositing the PEDOT layer into a N_2-filled glove box. The PEDOT solution was spin coated onto ITO substrates at 4000 rpm for 30 s, and then baked on a hotplate at 150 °C for 10 min. After annealing, the ITO/PEDOT substrates were allowed to cool down to room temperature naturally. Subsequently, a thin layer of Al_2O_3 was deposited on top of PEDOT, as reported in a previous study [13]. Al_2O_3 nanoparticles dispersion (Sigma-Aldrich) was diluted with IPA and spin-coated onto ITO/PEDOT substrates at 4500 rpm for 30 sec and annealed for 3 minutes at 100°C.

Preparation of perovskite layer. A solution of $Cs_{0.15}FA_{0.85}SnI_{1.5}Br_{1.5}$ (1 M) was prepared by mixing 0.15 M CsI (Alfa- Aesar, 99.999%), 0.85 M FAI (GreatCell Solar), 0.25 M SnI_2 (Sigma-Aldrich, 99.99%) and 0.75 M $SnBr_2$ (TCI, 97%) in anhydrous DMF: DMSO 4:1 (v:v). The precursor solution was spin-coated onto ITO/PEDOT/Al_2O_3 substrates at 4000 rpm for 50 seconds. After 25 seconds, 120 μL of anhydrous anisole was casted onto the substrates. The films were placed on a hotplate at 100 °C for 30 min. The DMSO-free solar cells were prepared by using $FASn(I_{0.5}Br_{0.5})_3$ and $FASn(I_{0.67}Br_{0.33})_3$. 1.2 M Sn-based mixed-halide perovskite solution was obtained by mixing varying ratios of equimolar solutions as mentioned above apart from solvent system. N,N-Diethylformamide (DEF):1,3-Dimethyl-3,4,5,6-tetrahydro-2(1*H*)-pyrimidinone (DMPU) (6:1, v:v) solvent mixture is utilized [13]. The perovskite precursor (with 10% over-stoichiometry of SnI_2 and 5% $EDAI_2$) was spun at 500 rpm for 5 s and 4000 rpm for 25 seconds.

100 μL of DEE were dropped on the spinning substrate at 21 s from the start of the deposition process. The film was annealed at 100 °C for 30 minutes.

Preparation of hole transporting layer and Ag electrode. The electron-transport layer precursor was prepared by dissolving 20 mg PCBM (phenyl-C61-butyric acid methyl ester) in 1 mL of chlorobenzene. The precursor was then spin-coated on the perovskite layer at 2000 rpm for 30 s. A thin Bathocuproine (BCP) buffer layer was deposited on top of PCBM by spin coating a BCP solution (0.5 mg/mL in IPA) at 4000 rpm for 30 seconds. Finally, 75 nm of Ag was deposited under a vacuum of $<1\times10^{-6}$ mbar with a rate of 0.1-0.6 nm/s. The preparation of the Ag electrode was performed in a N_2-purged glovebox. The DMSO-free solar cells were prepared by evaporating a C_{60} layer (40 nm) as electron selective layer and BCP (9 nm) as buffer layer at a 10^{-6} mbar vacuum level with a deposition rate between 0.1 and 0.3 Å/s, respectively. Finally, a 120 nm thick layer of silver was thermally evaporated as the top electrode.

Supplementary Figures

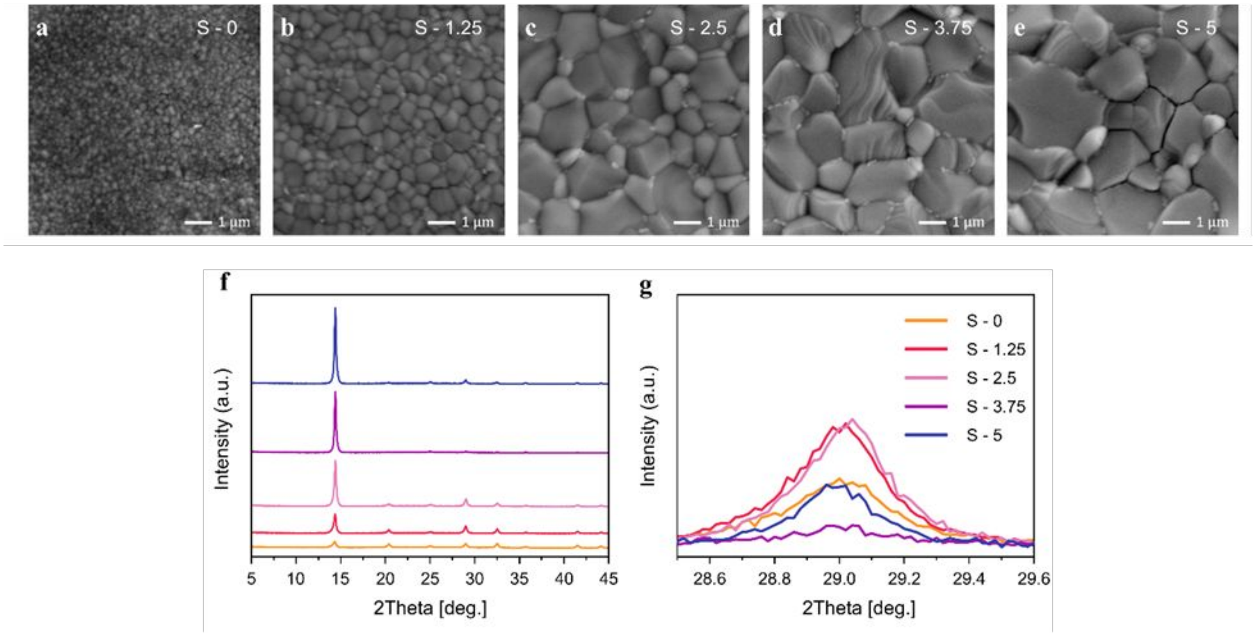

Figure S1 | a-e, SEM images of $Cs_{0.17}FA_{0.83}Pb(I_{0.5}Br_{0.5})_3$ perovskites prepared with 0, 1.25, 2.5, 3.75, 5 mol% of $Pb(SCN)_2$-containing additive (nomenclature: S – molar ratio of $Pb(SCN)_2$-containing additive). f, XRD patterns of perovskite films prepared from $Pb(SCN)_2$-contained additive with mole ratio ranging from 0 to 5 mol%. g, (002) peaks of the XRD patterns.

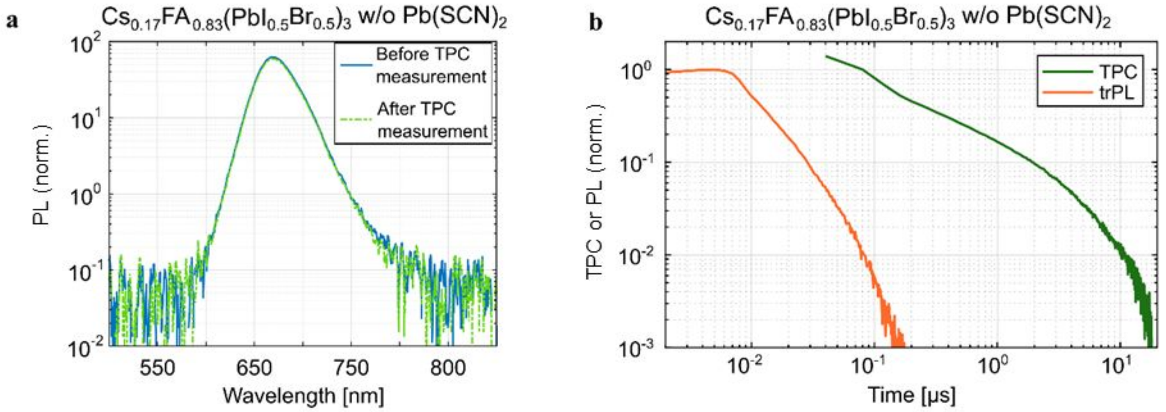

Figure S2 | a, PL spectra of Pb mixed-halide (without $Pb(SCN)_2$) before and after the transient photocurrent (TPC) measurement. b, Normalized transient photocurrent (TPC) and time-resolved photoluminescence (TRPL) kinetics.

Discussion | Due to the short duration of the measurements (a few seconds) and the low repetition rate (1 kHz) of the pulsed excitation light, we don't observe signs of halide segregation (rise of a red-shifted secondary PL peak) from the recorded spectra shown in Fig. S2. Although TPC cannot resolve the fast sub-microsecond dynamics, we observe that between 1 and 10 μs carriers are not recombining radiatively; hence the long-lived free carrier tail that generates the TPC signal can be ascribed to a residual population of either only free electron or holes, resulting from the selective trapping of their respective opposite charged counterparts.

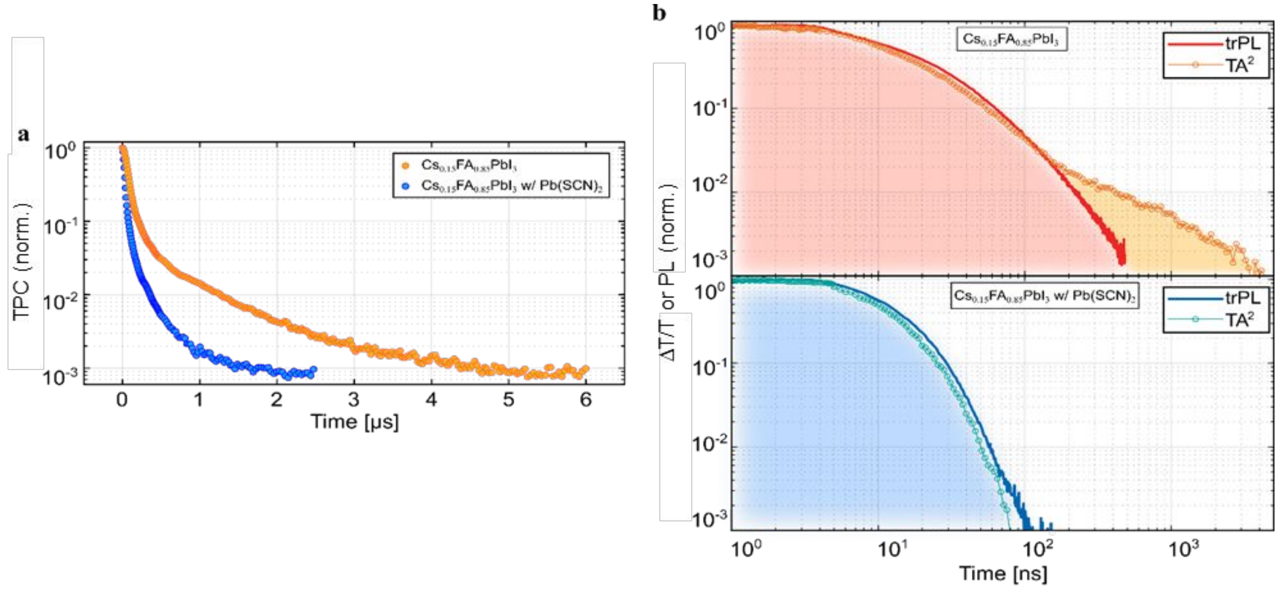

Figure S3 | a, Transient photocurrent (TPC) of $Cs_{0.17}FA_{0.83}PbI_3$ with and without the addition of $Pb(SCN)_2$. b, Transient absorption (TA) and TRPL of pure iodide samples prepared without (0 mol%) and with 5 mol% $Pb(SCN)_2$ additive.

Discussion | Fig. S3 shows how the $Pb(SCN)_2$ additive affects the photo-physics of $Cs_{0.17}FA_{0.83}PbI_3$ perovskites, observing the same phenomenology reported for $Cs_{0.17}FA_{0.83}Pb(I_{0.5}Br_{0.5})_3$.

The long-lived tail of the TPC is lifted by the addition of $Pb(SCN)_2$. Since the single halide perovskite does not present halide segregation and the spectral instabilities, we could perform a more detailed spectroscopy analysis that required a longer illumination time than the techniques we applied to mixed halides samples. Indeed, since the long lived photocurrent in pristine samples represents the presence of a long lived free carrier population, this should be observable as a bleach at the perovskite band edge due to state filling in the valance or conduction band. By overlapping the TRPL kinetics and the squared TA kinetics (see methods), we can observe that in the pristine sample, the two kinetics diverge, in fact, the TA kinetic follows a single-charge population of free carriers that cannot recombine radiatively since the long-lived carrier trap withhold their counterparts. The same effect is not observed in the 5 mol% sample, which indicates such long-lived traps are not present in sample prepared with $Pb(SCN)_2$-contained additive.

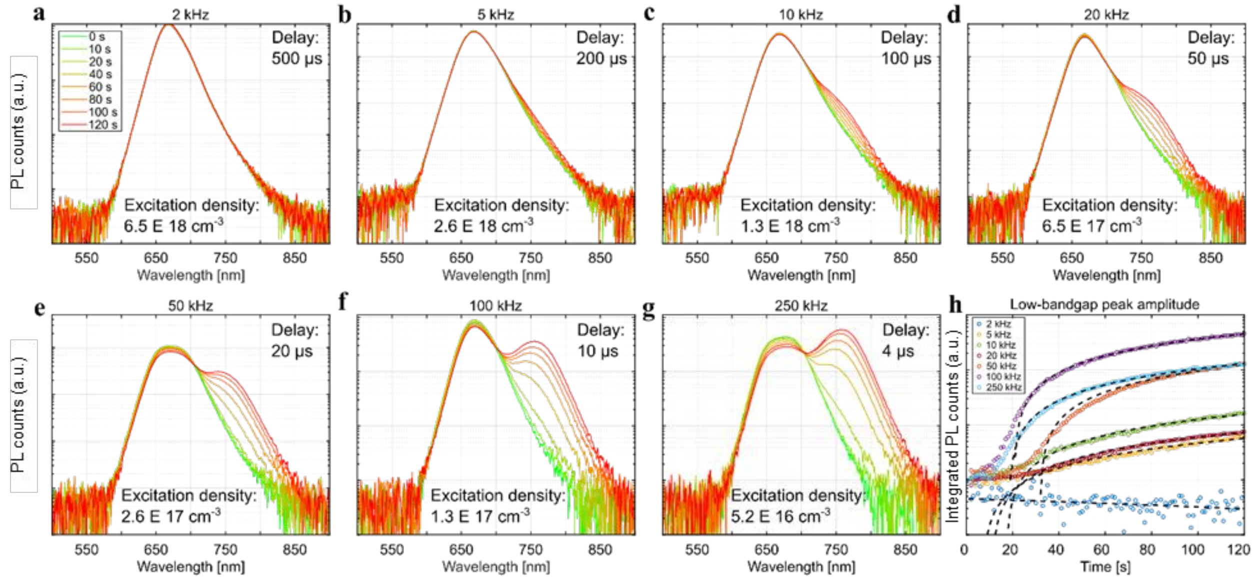

Figure S4 | a-g, Photoluminescence (PL) spectra of $Cs_{0.17}FA_{0.83}Pb\ (I_{0.5}Br_{0.5})_3$ without the $Pb(SCN)_2$ additive at an increasing repetition rate of the excitation pulsed femtosecond laser (Pharos). h, Integrated amplitude of the gaussian component of the fit function, representing the growth of the segregated phase in S-0 under pulsed light at different inter-pulse duration (constant fluence). We apply a linear fit $a*t + b$ to these dynamics to extract the rate of halide segregation (defined as $1/a$) displayed in Fig. 3d in the main text.

Discussion | The fluence is kept constant over the measurements shown in Figs. S4a-g to rule out that the observed halide segregation at a high repetition rate could depend on the increased optical power delivered to the material. Consequently, the pulse energy and excitation density decrease proportionally with the increasing repetition rate. A custom function defined as the sum of an ex-gaussian and a gaussian is used to fit each spectrum. The ex-gaussian fits the PL peak of the main phase of the material at 670 nm (and accommodates emission from lower bandgap states given by disorder). The gaussian component fits the PL emission from the lower-bandgap segregated phase.

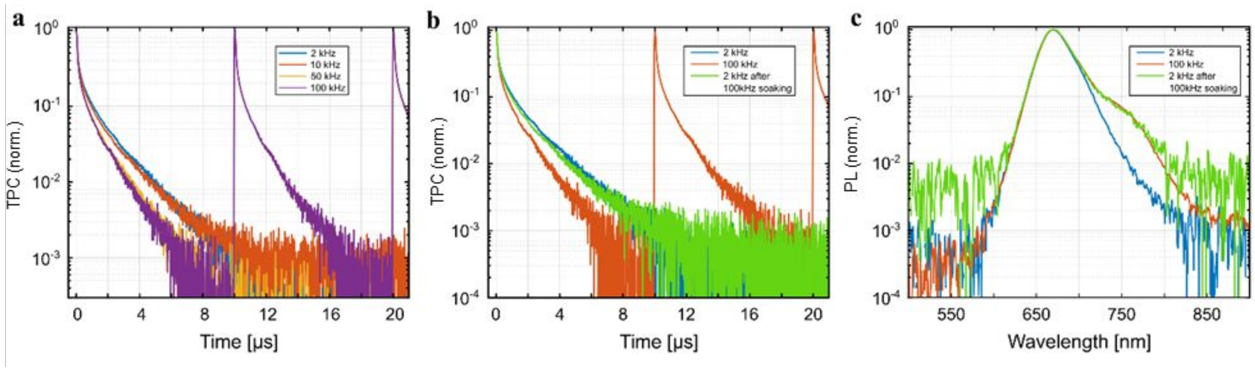

Figure S5 | a, Transient photocurrent (TPC) kinetics of $Cs_{0.17}FA_{0.83}Pb\ (I_{0.5}Br_{0.5})_3$ prepared without $Pb(SCN)_2$ at increasing repetition rate of the pulsed excitation light (Pharos laser, 515 nm, excitation density of 1E18 cm^{-3} constant for all the measurements to ensure the same signal-to-noise ratio, being TPC solely dependent on pulse energy). b TPC and c PL measurements of $Cs_{0.17}FA_{0.83}Pb\ (I_{0.5}Br_{0.5})_3$ prepared without $Pb(SCN)_2$ taken with the excitation laser set at 2 kHz (inter-pulse delay of 500 µs), displayed with blue lines in both graphs.

Discussion | The long-lived free carrier tail shown in Fig. S5a decreases in length at a repetition rate of 50 kHz (inter-pulse delay of 20 µs). This effect may seem counter-intuitive since, with other techniques such as trPL and TA, when the delay between pulses becomes comparable with the duration of the kinetics, carrier pile-up effects are observed, with an increase of the length and amplitude of tail signals due to the accumulation of charge carriers over repeated excitation cycles. What we believe is causing the shortening of the tails is a similar process that instead involves trapped carriers. This dark-pile up effectively lowers the availability of long-lived traps that are already partially filled when the light pulse reaches the sample, due to the effect of previous pulses. Since the long-lived traps are temporarily healed, carriers will recombine through other paths (radiative recombination or fast traps). This also helps us speculate that the lifetime of the trapped carriers might be even slightly longer than the measured free carriers in TPC. When the dark-pile up takes place, a fraction of the carrier is always trapped (quasi-CW regime), favouring reactions between filled traps.
Figs. S5b-c shows the simultaneous TPC and PL measurements to demonstrate that the shortening of the TPC tails is not caused by halide segregation. With the excitation used to take these measurements (excitation density 1E18 cm^{-3}), we do not observe the effect of halide segregation in PL over extended period of time (several minutes). We quickly recorded TPC and PL at 100 kHz (inter-pulse delay of 10 µs) (orange). Since the pulse energy is preserved, the fluence increases considerably, and the material quickly segregates during the measurements. By rapidly repeating TPC and PL at 2kHz after the last acquisition, we can still observe the effect of halide segregation in PL, with a lower bandgap emission peak (green), while the TPC tail is perfectly comparable to the initial blue tail. Hence, the length of the TPC is not heavily influenced by the presence of a segregated phase. This helps us confirm the previous repetition rate-dependent analysis. We also expect an even lower impact of halide segregation in the previous analysis, due to the lowering of the excitation density at a higher repetition rate (the laser fluence was kept constant between the measurements).

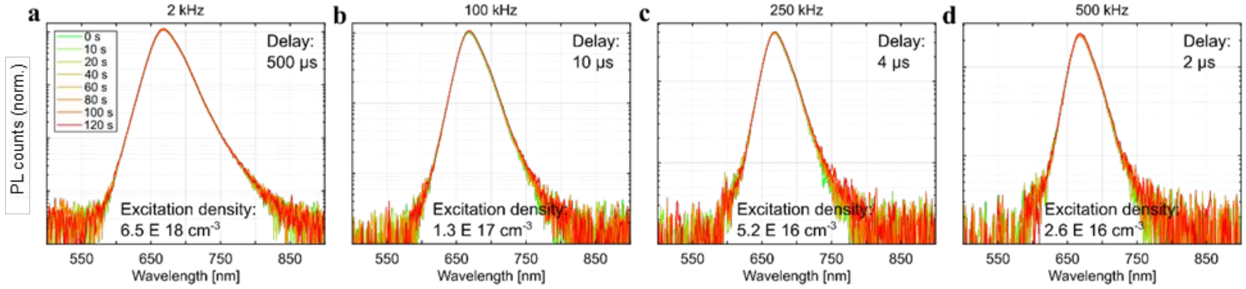

Figure S6 | a-d, Photoluminescence (PL) spectra of $Cs_{0.17}FA_{0.83}Pb(I_{0.5}Br_{0.5})_3$ prepared with 5 mol% $Pb(SCN)_2$-containing additive (S-5).

Discussion | We cannot observe any sign of halide segregation even at higher repetition rates when the inter-pulse delay becomes 2 μs (500 kHz).

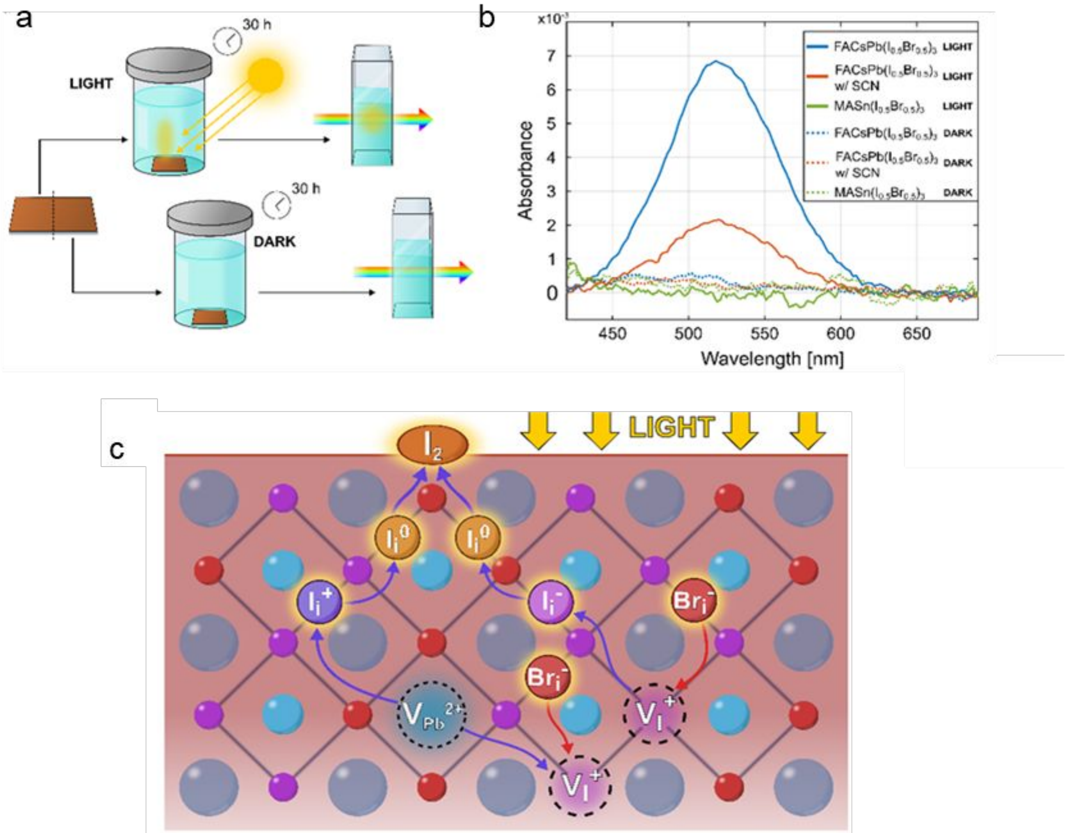

Figure S7 | a, Schematics of the I_2 expulsion experiments (see Methods for more details). b, UV-Vis spectra of the toluene submerging the perovskites during the I_2 expulsion experiment. $Cs_{0.17}FA_{0.83}Pb(I_{0.5}Br_{0.5})_3$ prepared with addition of $Pb(SCN)_2$ (red) shows reduced I_2 expulsion under illumination compared to the reference material (blue). $MASn(I_{0.5}Br_{0.5})_3$ (green) doesn't release detectable traces of I_2 under illumination for the whole duration of the experiment. Dark spectra for each material are displayed in dashed lines. c, Scheme summarizing the de-mixing process in $MAPb(I_{0.5}Br_{0.5})_3$.

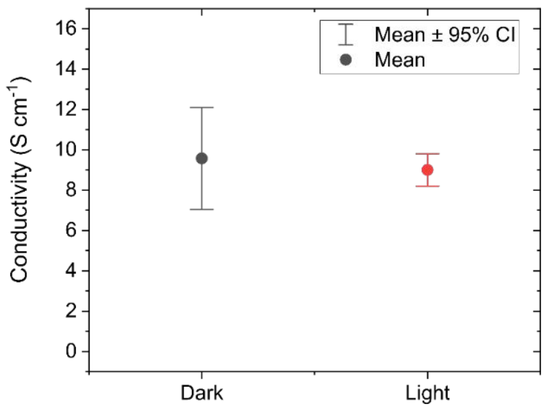

Figure S8 | Conductivity of $FACsSnI_3$ thin films before and after light soaking for 180 s.

Discussion | The conductivity is measured in the same spot in the dark and after 180 seconds of illumination (illumination source: LED lamp, 3 W, RS Pro), Showing that illumination does not alter the conductivity of the film.

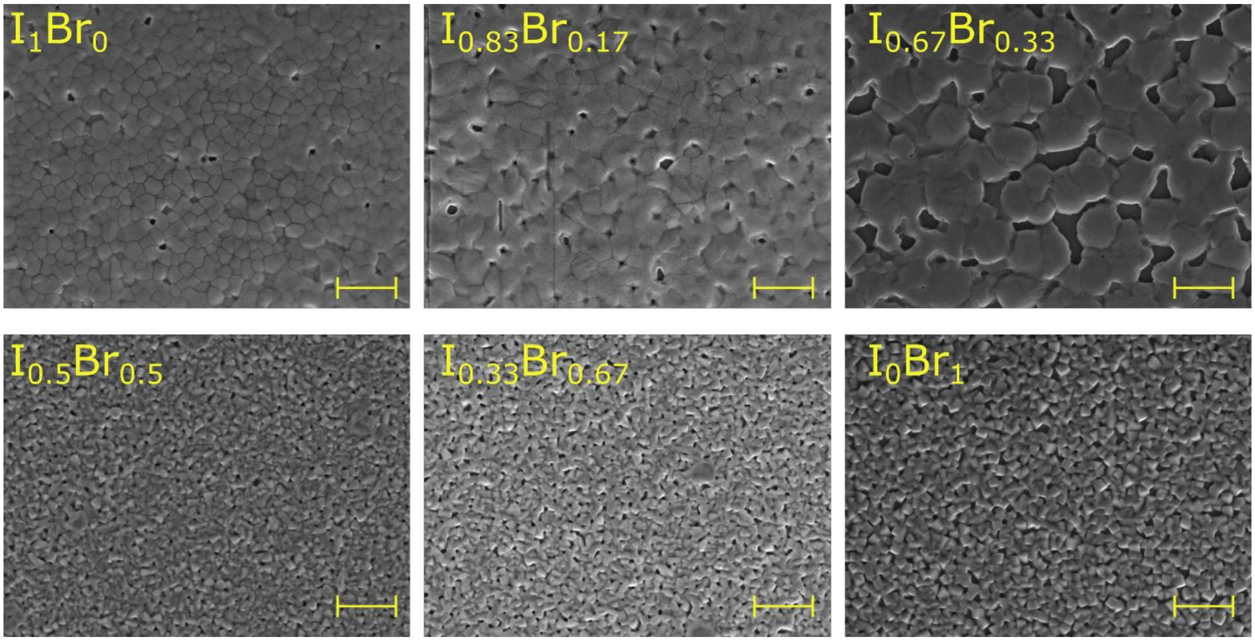

Figure S9 | Top-view SEM images of $MASn(I_{1-x}Br_x)_3$ thin films with x=0, 0.17, 0.33, 0.5, 0.67 and 1. Scale bar is 2 µm.

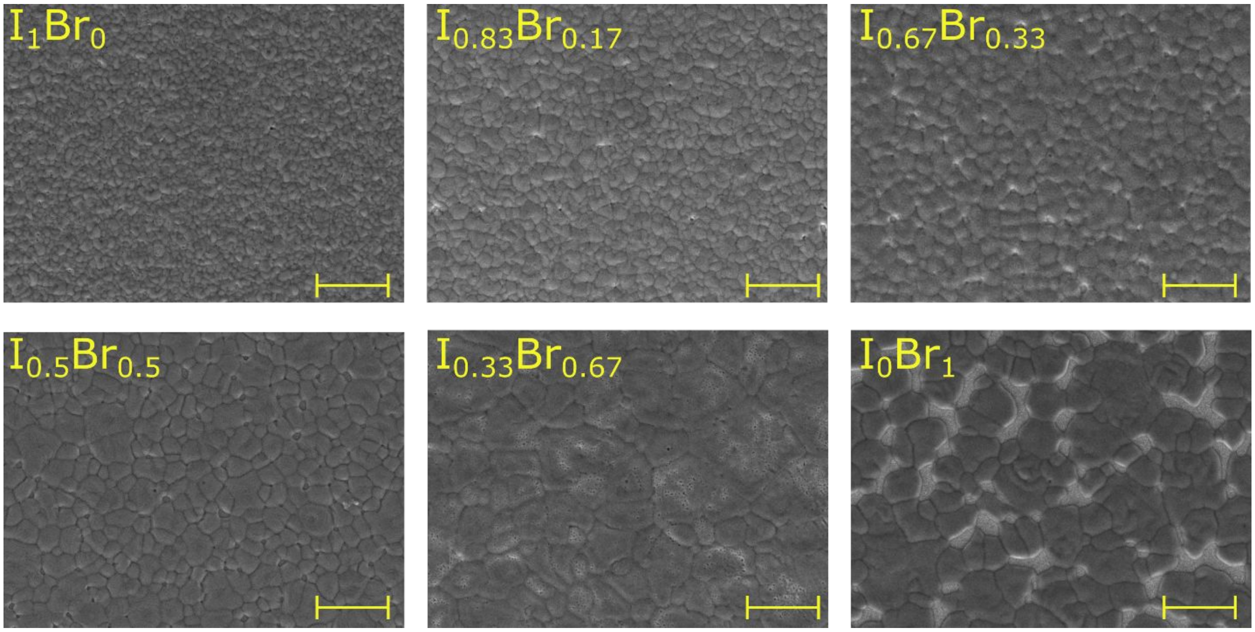

Figure S10 | Top-view SEM images of $FASn(I_{1-x}Br_x)_3$ thin films with x=0, 0.17, 0.33, 0.5, 0.67 and 1. Scale bar is 5 µm.

Discussion | SEM top-view images showed in Figs. S9 and S10 highlight that the morphology is affected by the cation. Overall, MA-based Sn perovskites present higher pin-holes density when compared to the FA-based Sn perovskite thin films. The addition of Br in FA-based perovskite

thin films causes an enlargement of the grain size. In contrast, in MA-based perovskites, the grain enlargement is observed only for Br addition up to Br percentages of 33%, while for Br contents higher than 50% reduced grain size is observed. Importantly, we note that perovskite films shown in Figs. S9 and S10 are pristine films, i.e., prepared with no additives, and that their fabrication process has not been optimized. Despite the non-perfect uniformity of the films, all materials are extremely photostable, whichever Br/I ratio is used. In order to obtain specific grain size and uniformity of the films, each composition should be carefully optimized through an in-depth understanding and rational control of film-forming dynamics, which is out of the scope of this paper.

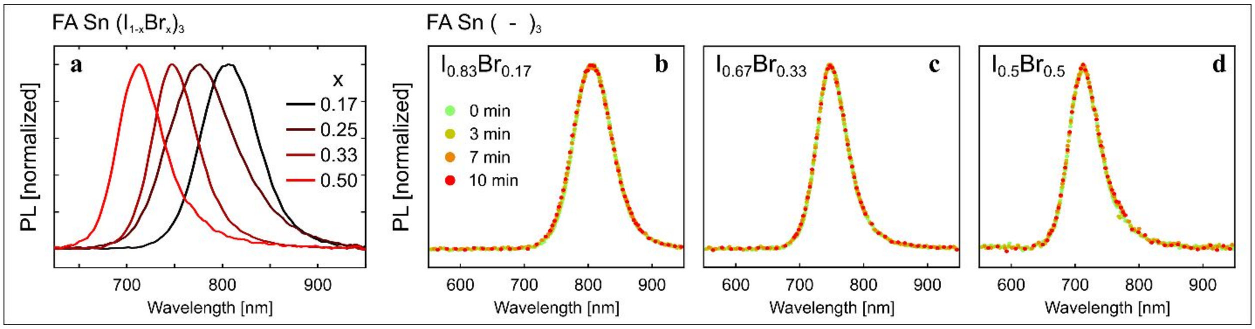

Figure S11 | a, Steady-state photoluminescence (PL) spectra of FA Sn $(I_{1-x}Br_x)_3$ perovskites at increasing Br content. b-d, Photoluminescence (PL) monitored under 1 Sunlight soaking of FA-based perovskites with increasing Br content. For each sample, the PL spectrum doesn't change during the duration of the experiment.

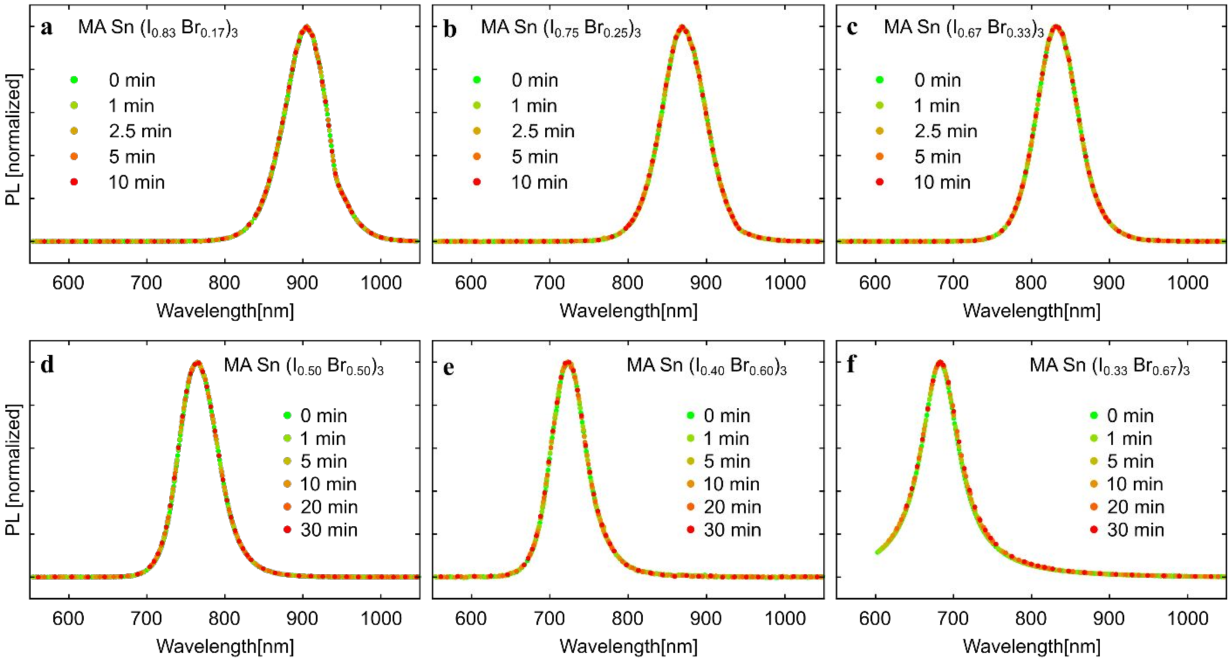

Figure S12 | a-f, Photoluminescence (PL) stability test at 1 Sun illumination (50 mW/cm^2, 450 nm diode laser) of MA Sn $(I_{1-x}Br_x)_3$ perovskites with various I : Br ratios. No effect of halide segregation can be observed even at high Br content and extended light soaking duration.

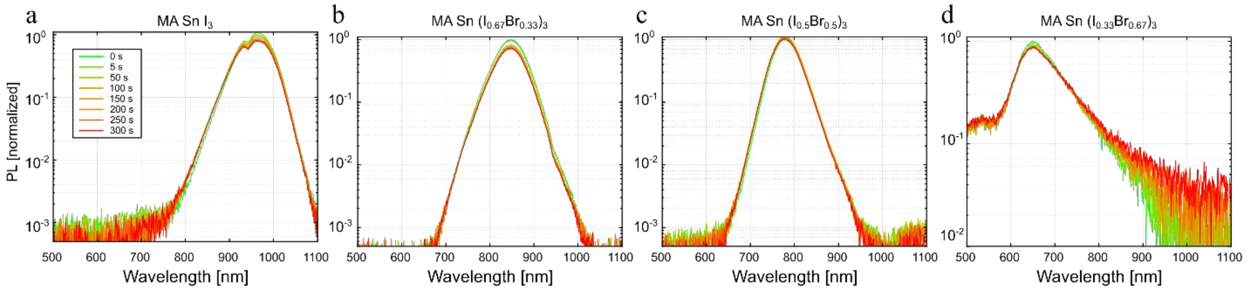

Figure S13 | a-d, Photoluminescence (PL) stability test at 10 Sun illumination (500 mW/cm^2, 450 nm diode laser) of MA Sn $(I_{1-x}Br_x)_3$ perovskites at various I : Br ratios. No effect of halide segregation can be observed for all samples.

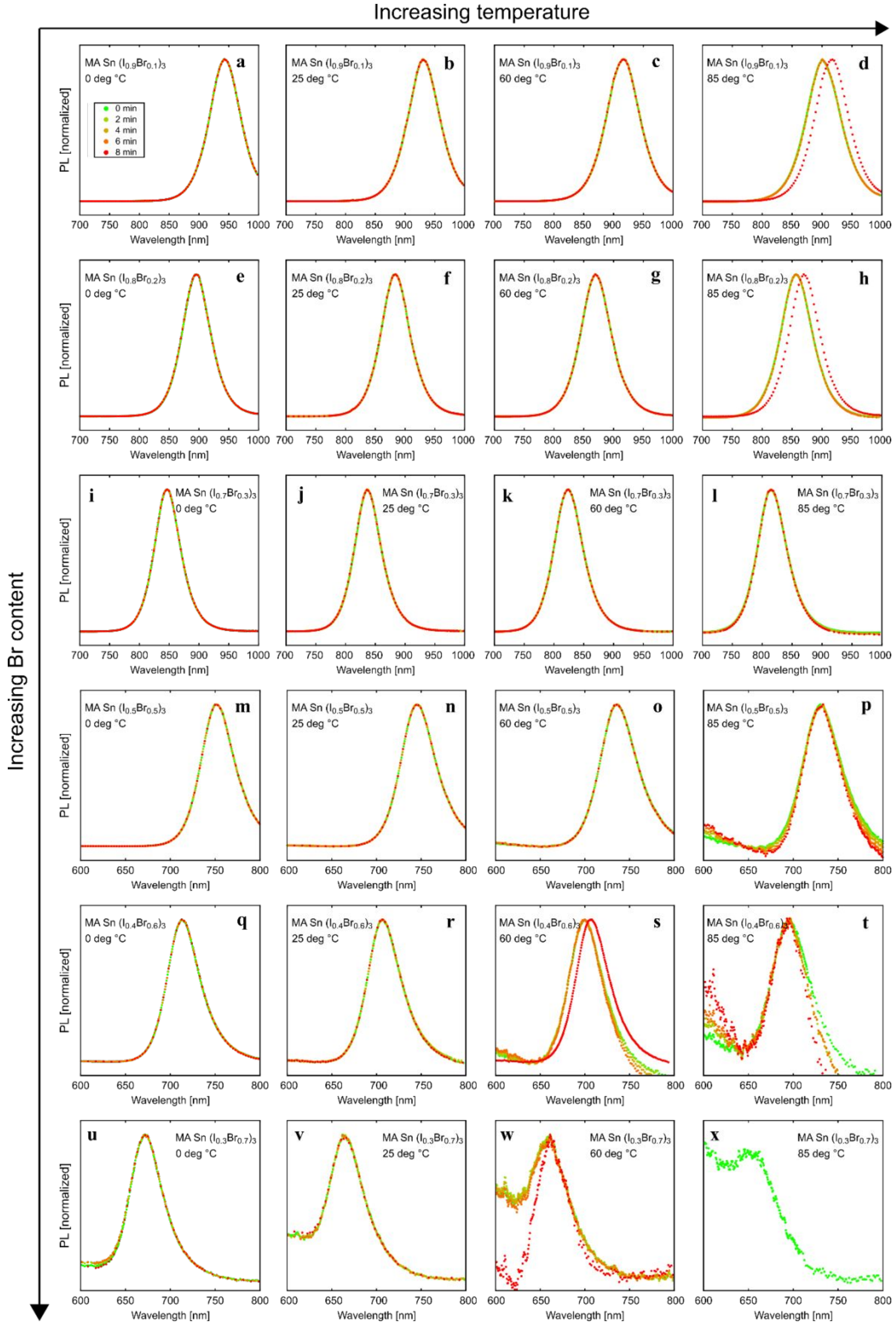

Figure S14 | a-x, Photoluminescence (PL) spectra recorded under 30 mW/cm^2 continuous excitation light (530 nm diode laser).

Discussion | Few samples showed instability after 8 minutes of light soaking (Figs. S14d, S14h and S14s), although without developing a second red-shifted peak as commonly observed in A-Pb($I_{1-x}Br_x$)$_3$ perovskites (PL from lower bandgap segregated phase). Instead, the rigid shift of the main PL peak might be connected to the film degradation at high temperatures. Moreover, this effect doesn't affect only potentially more unstable Br rich samples but also those with a low Br concentration. The sporadic showing of changes in the PL spectra from this set of measurement made us disregard the possibility for thermally activated halide segregation in $MASn(I_{1-x}Br_x)_3$ perovskites. At high temperature, $MASn(I_{0.3}Br_{0.7})_3$ shows in Fig. S14x such a weak PL emission that prevented the collection of clear spectra.

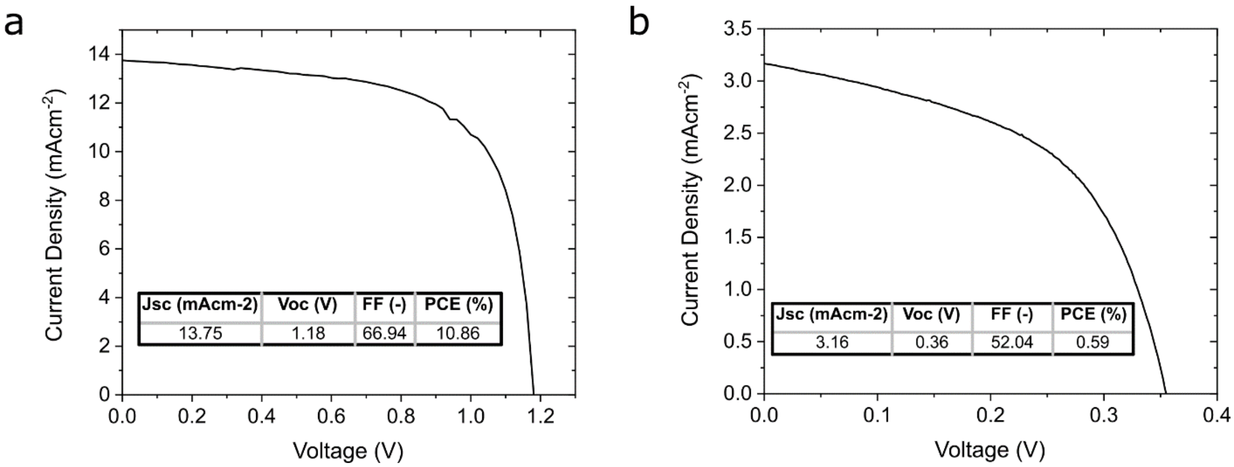

Figure S15 | a, Current density-voltage curve of a $FA_{0.83}Cs_{0.17}PbI_{1.5}Br_{1.5}$ solar cell with a FTO/SnO_2/perovskite/spiro-OMeTAD/Au configuration. b, Current density-voltage curve of a $FA_{0.83}Cs_{0.17}SnI_{1.5}Br_{1.5}$ solar cell with an ITO/PEDOT/Al_2O_3/perovskite/PCBM/BCP/Ag configuration (note that this solar cell has been fabricated with pristine Sn perovskite materials, i.e., without any additives).

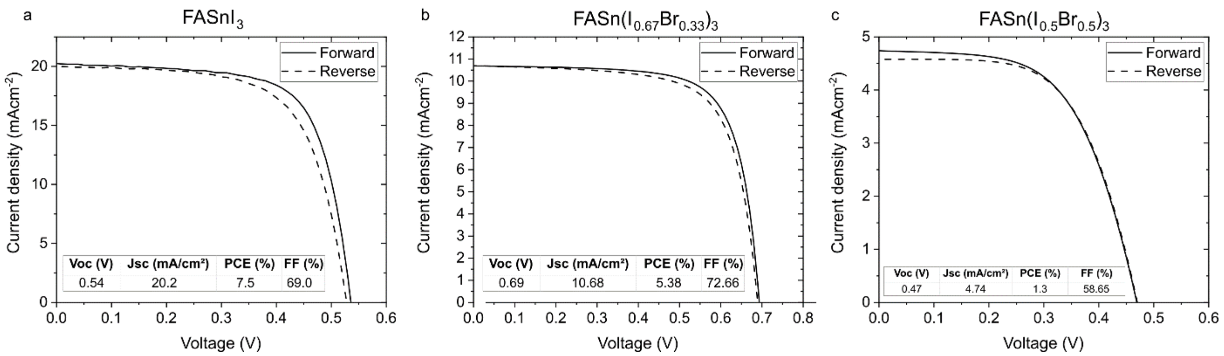

Figure S16 | Current density-voltage curve of a) $FASnI_3$, b) $FASn(I_{0.67}Br_{0.33})_3$ and c) $FASn(I_{0.5}Br_{0.5})_3$ solar cells fabricated with 5 mol% $EDAI_2$ with a ITO/PEDOT/Al_2O_3/perovskite/C60/BCP/Ag configuration, measured under simulated 1 sun AM1.5G illumination. The table lists the main photovoltaic parameters of the solar cell under forward scan.

Discussion | The use of additives within the perovskite precursor solution can limit the Sn^{2+}/Sn^{4+} oxidation, slow down the crystallization and passivate trap defects, improving the photovoltaic conversion efficiency.[14, 15] Here, we use 5 mol% of ethylenediammonium diiodide ($EDAI_2$) as additive and observe power conversion efficiencies of ~1.3% and ~5.5% on $FASn(I_{0.5}Br_{0.5})_3$ and $FASn(I_{0.67}Br_{0.33})_3$ solar cells, respectively.

Supplementary Discussion

SD1. Thermodynamic stability of the bulk mixed halide perovskites

The stability of mixed halide perovskites has been investigated for different I/Br ratios in the DFT framework by calculating the associated mixing free energies with respect to the full lead and tin perovskites. We modelled mixed $MAPb(I_{1-x}Br_x)_3$ and $MASn(I_{1-x}Br_x)_3$ perovskites starting from the pristine tetragonal $MAPbI_3$ and $MASnI_3$ phases by replacing I ions with Br to obtain different I/Br ratios, i.e. x = 0, 0.16, 0.33, 0.50, 0.66, 0.84, 1. Cell parameters and ion positions have been relaxed in all cases by using the PBE functional [10] and including DFT-D3 dispersion interactions [4]. DFT calculations have been performed with the Quantum Espresso simulation package [16] by using ultrasoft pseudopotentials with a cutoff on the wavefunctions of 40 Ry (320 Ry on the charge density) and 4x4x4 k-point grids in the Brillouin zone (BZ). By moving from the pure iodide to the pure bromide phases a shrinking of the lattice and an increase of the electronic band gap is observed, see Table S2 and Table S3. We monitored the stability of the mixed perovskites by calculating the mixing Gibbs free energies at room temperature starting from full lead and tin perovskites,

$$\Delta G(x,T)_{mix} = \Delta E_{mix}(x) - T\Delta S_{mix}(x) \quad \text{(eq. 1)}$$

Where

$$\Delta E_{mix}(x) = E_{MASn/Pb(I_{1-x}Br_x)_3} - (1-x)E_{MASn/PbI_3} - xE_{MASn/PbBr_3} \quad \text{(eq. 2)}$$

is the mixing energy at 0 K, calculated as the difference of the total energies of the mixed I/Br phase and the full lead and tin phases; and the entropy of mixing is approximated by the expression

$$\Delta S(x) = -k_B\,[x\,ln\,x + (1-x)ln\,(1-x)] \quad \text{(eq. 3)}$$

Results are reported in Table S2 and Table S3. The mixing energy at 0 K, i.e. ΔE_{mix}, is positive in all cases, while the inclusion of the entropy of mixing stabilizes the phases at room temperature. Non-linear behavior of the mixing Gibbs free energies with I/Br ratio x is observed, with values up to 30 meV/f.u. for low Br contents, in agreement with previous studies [17].

Table S2. Optimized lattice parameters, volumes, band gaps (E_g), mixing energies (ΔE_{mix}) and mixing Gibbs free energies (ΔG_{mix}) at 300K of the modelled mixed $MAPb(I_{1-x}Br_x)_3$ phases for different I/Br ratios.

	a (Å)	b (Å)	c (Å)	Volume ($Å^3$)	E_g (eV)	ΔE_{mix} (eV/f.u.)	$\Delta G_{mix}(300K)$ (eV/f.u.)
$MAPbI_3$	8.774	8.737	12.942	3543	1.49	-	-

$MAPb(I_{0.84}Br_{0.16})_3$	8.788	7.766	12.555	3454	1.62	0.00	-0.03
$MAPb(I_{0.66}Br_{0.33})_3$	8.688	8.624	12.578	3365	1.66	0.03	-0.02
$MAPb(I_{0.5}Br_{0.5})_3$	8.543	8.479	12.564	3250	1.73	0.03	-0.02
$MAPb(I_{0.33}Br_{0.66})_3$	8.418	8.374	12.568	3164	1.77	0.03	-0.01
$MAPb(I_{0.16}Br_{0.84})_3$	8.309	8.256	12.554	3076	1.82	0.02	-0.01
$MAPbBr_3$	8.297	8.248	12.168	2974	1.95	-	-

Table S3. Optimized lattice parameters, volumes, band gaps (E_g), mixing energies (ΔE_{mix}) and mixing Gibbs free energies (ΔG_{mix}) at 300K of the modelled mixed $MASn(I_{1-x}Br_x)_3$ phases for different I/Br ratios.

	a (Å)	b (Å)	c (Å)	Volume (Å^3)	E_g (eV)	ΔE_{mix} (eV/f.u.)	$\Delta G_{mix}(300K)$ (eV/f.u.)
$MASnI_3$	8.712	8.699	12.636	3420	0.47	-	-
$MASn(I_{0.84}Br_{0.16})_3$	8.712	8.684	12.295	3330	0.62	0.01	-0.03
$MASn(I_{0.66}Br_{0.33})_3$	8.642	8.578	12.333	3265	0.65	0.03	-0.02
$MASn(I_{0.5}Br_{0.5})_3$	8.475	8.442	12.328	3150	0.70	0.02	-0.03
$MASn(I_{0.33}Br_{0.66})_3$	8.392	8.374	12.334	3095	0.82	0.04	-0.01
$MASn(I_{0.16}Br_{0.84})_3$	8.292	8.232	12.311	3001	0.84	0.02	-0.01
$MASnBr_3$	8.220	8.199	11.927	2870	0.89	-	-

SD2. Stability of the mixed halide perovskite surfaces

The thermodynamic stability of the mixed $MAPb(I_{0.5}Br_{0.5})_3$ surface has been analyzed by calculating the surface formation energies (SFE) of the (001) MAI-terminated and half-MAI-terminated surfaces. The SFE has been calculated by following the expression

$$SFE = \frac{E^S - \sum_i n_i \mu_i}{2A}$$

Where E^S is the energy of the surface slab, n and μ are the number of ion species in the slab and the relative chemical potentials, and A is the area of the surface slab. Chemical potentials of the species have been set following the same approach used in halide defects calculations. Calculations have been performed in the 2x2 in-plane slabs within the supercell approach by using the CP2K code and the same computational setup used for I_2 molecule and halide defect couple calculations at the surface, described in the main text (PBE-D3, GTH pseudopotentials, DZVP basis set, density cutoff 300 Ry). Results are reported in Table S4.

Table S4. Calculated SFE (eV/nm^2) of the MAI-terminated and half MAI-terminated (001) surfaces of $MAPb(I_{0.5}Br_{0.5})_3$, compared with the calculated values in the pure $MAPbI_3$ and $MAPbBr_3$ phases.

phase	(001) MAI-terminated	(001) – Half MAI-terminated
$MAPb(I_{0.5}Br_{0.5})_3$	0.41	1.18
$MAPbI_3$	0.15	1.08
$MAPbBr_3$	0.14	1.04

As reported in Table S4, SFEs for the mixed $MAPb(I_{0.5}Br_{0.5})_3$ perovskite are higher than for the pure $MAPbI_3$ and $MAPbBr_3$ phases. This suggests that in proximity of the surface a redistribution of halide ions may take place to reduce the surface energy. To illustrate this concept, the energy of the MAI-terminated (001) surface of $MAPb(I_{0.5}Br_{0.5})_3$ has been recalculated by exchanging two halide ion couples between the bulk and the surface layers in order to create slabs with an inhomogeneous distribution of halides at the surface and in the center of the slab (see Fig. S17). The analysis of the relative energies indicates that slabs with an I-rich (I-poor) surface layer (bulk), Fig. S17c, are more stable than slabs with a homogeneous distribution of halides, Fig. S17a. The opposite is observed by accumulating Br ions at the surface layers.

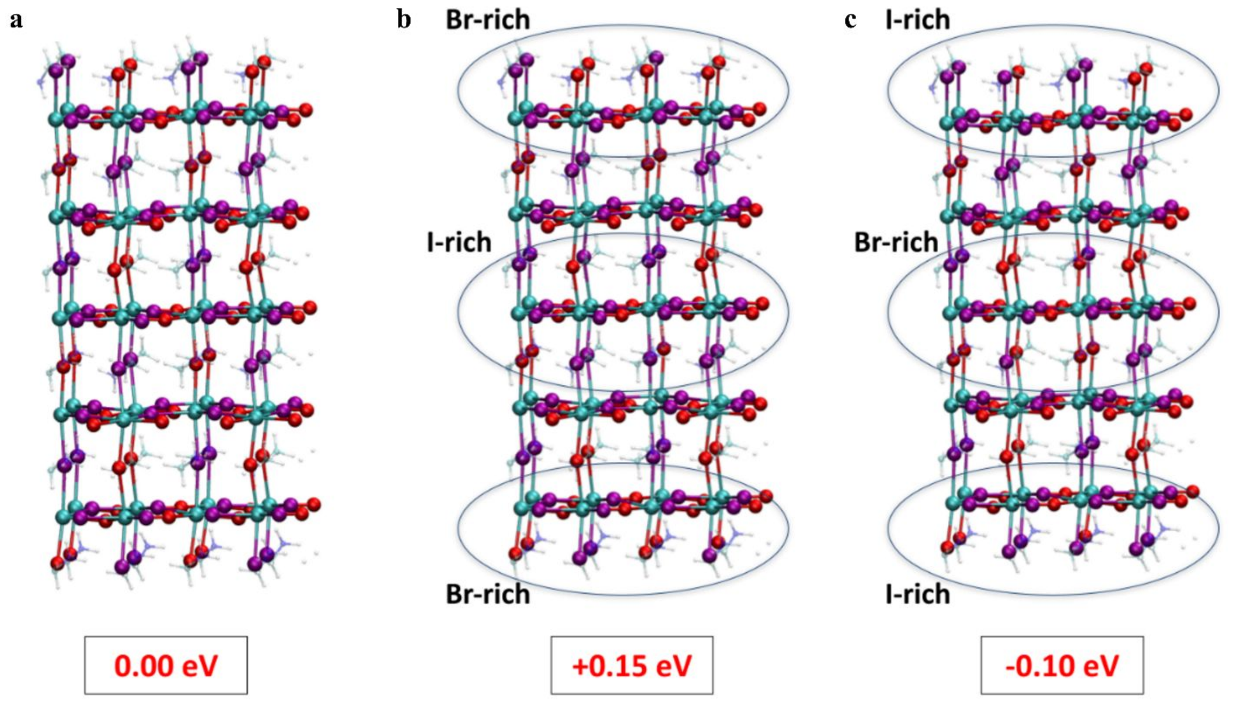

Figure S17 | With a, homogeneous and b-c, inhomogeneous distributions of halide ions between the bulk and the surface layers, along with the relative total energies.

References:

(1) Leijtens, T.; Eperon, G. E.; Barker, A. J.; Grancini, G.; Zhang, W.; Ball, J. M.; Kandada, A. R. S.; Snaith, H. J.; Petrozza, A. Carrier trapping and recombination: the role of defect physics in enhancing the open circuit voltage of metal halide perovskite solar cells. *Energy & Environmental Science* **2016**, *9* (11), 3472-3481, 10.1039/C6EE01729K. DOI: 10.1039/C6EE01729K.

(2) VandeVondele, J.; Krack, M.; Mohamed, F.; Parrinello, M.; Chassaing, T.; Hutter, J. Quickstep: Fast and accurate density functional calculations using a mixed Gaussian and plane waves approach. *Computer Physics Communications* **2005**, *167* (2), 103-128. DOI: https://doi.org/10.1016/j.cpc.2004.12.014.

(3) Perdew, J. P.; Ernzerhof, M.; Burke, K. Rationale for mixing exact exchange with density functional approximations. *The Journal of Chemical Physics* **1996**, *105* (22), 9982-9985. DOI: 10.1063/1.472933.

(4) Grimme, S.; Antony, J.; Ehrlich, S.; Krieg, H. A consistent and accurate ab initio parametrization of density functional dispersion correction (DFT-D) for the 94 elements H-Pu. *The Journal of Chemical Physics* **2010**, *132* (15), 154104. DOI: 10.1063/1.3382344.

(5) Goedecker, S.; Teter, M.; Hutter, J. Separable dual-space Gaussian pseudopotentials. *Physical Review B* **1996**, *54* (3), 1703-1710. DOI: 10.1103/PhysRevB.54.1703.

(6) VandeVondele, J.; Hutter, J. Gaussian basis sets for accurate calculations on molecular systems in gas and condensed phases. *The Journal of Chemical Physics* **2007**, *127* (11), 114105. DOI: 10.1063/1.2770708.

(7) Guidon, M.; Hutter, J.; VandeVondele, J. Auxiliary Density Matrix Methods for Hartree−Fock Exchange Calculations. *Journal of Chemical Theory and Computation* **2010**, *6* (8), 2348-2364. DOI: 10.1021/ct1002225.

(8) Komsa, H.-P.; Rantala, T. T.; Pasquarello, A. Finite-size supercell correction schemes for charged defect calculations. *Physical Review B* **2012**, *86* (4), 045112. DOI: 10.1103/PhysRevB.86.045112.

(9) Freysoldt, C.; Grabowski, B.; Hickel, T.; Neugebauer, J.; Kresse, G.; Janotti, A.; Van de Walle, C. G. First-principles calculations for point defects in solids. *Reviews of Modern Physics* **2014**, *86* (1), 253-305. DOI: 10.1103/RevModPhys.86.253.

(10) Perdew, J. P.; Burke, K.; Ernzerhof, M. Generalized Gradient Approximation Made Simple. *Physical Review Letters* **1996**, *77* (18), 3865-3868. DOI: 10.1103/PhysRevLett.77.3865.

(11) Yu, Y.; Wang, C.; Grice, C. R.; Shrestha, N.; Zhao, D.; Liao, W.; Guan, L.; Awni, R. A.; Meng, W.; Cimaroli, A. J.; et al. Synergistic Effects of Lead Thiocyanate Additive and Solvent Annealing on the Performance of Wide-Bandgap Perovskite Solar Cells. *ACS Energy Letters* **2017**, *2* (5), 1177-1182. DOI: 10.1021/acsenergylett.7b00278.

(12) Zhou, Y.; Jia, Y.-H.; Fang, H.-H.; Loi, M. A.; Xie, F.-Y.; Gong, L.; Qin, M.-C.; Lu, X.-H.; Wong, C.-P.; Zhao, N. Composition-Tuned Wide Bandgap Perovskites: From Grain Engineering to Stability and Performance Improvement. *Advanced Functional Materials* **2018**, *28* (35), 1803130, https://doi.org/10.1002/adfm.201803130. DOI: https://doi.org/10.1002/adfm.201803130 (acccessed 2022/05/11).

(13) Di Girolamo, D.; Aktas, E.; Ponti, C.; Pascual, J.; Li, G.; Li, M.; Nasti, G.; Alharthi, F.; Mura, F.; Abate, A. Enabling water-free PEDOT as hole selective layer in lead-free tin perovskite solar cells. *Materials Advances* **2022**, *3* (24), 9083-9089, 10.1039/D2MA00834C. DOI: 10.1039/D2MA00834C.

(14) Treglia, A.; Ambrosio, F.; Martani, S.; Folpini, G.; Barker, A. J.; Albaqami, M. D.; De Angelis, F.; Poli, I.; Petrozza, A. Effect of electronic doping and traps on carrier dynamics in tin halide perovskites. *Materials Horizons* **2022**, *9* (6), 1763-1773, 10.1039/D2MH00008C. DOI: 10.1039/D2MH00008C.
(15) Poli, I.; Ambrosio, F.; Treglia, A.; Berger, F. J.; Prato, M.; Albaqami, M. D.; De Angelis, F.; Petrozza, A. Photoluminescence Intensity Enhancement in Tin Halide Perovskites. *Advanced Science* **2022**, *9* (32), 2202795. DOI: https://doi.org/10.1002/advs.202202795.
(16) Giannozzi, P.; Baroni, S.; Bonini, N.; Calandra, M.; Car, R.; Cavazzoni, C.; Ceresoli, D.; Chiarotti, G. L.; Cococcioni, M.; Dabo, I.; et al. QUANTUM ESPRESSO: a modular and open-source software project for quantum simulations of materials. *Journal of Physics: Condensed Matter* **2009**, *21* (39), 395502. DOI: 10.1088/0953-8984/21/39/395502.
(17) Brivio, F.; Caetano, C.; Walsh, A. Thermodynamic Origin of Photoinstability in the CH3NH3Pb(I1–xBrx)3 Hybrid Halide Perovskite Alloy. *The Journal of Physical Chemistry Letters* **2016**, *7* (6), 1083-1087. DOI: 10.1021/acs.jpclett.6b00226.
